# Patient characteristics as effect modifiers for psoriasis biologic treatment response: an assessment using network meta-analysis subgroups

**DOI:** 10.1186/s13643-020-01395-6

**Published:** 2020-06-05

**Authors:** Ros Wade, Sahar Sharif-Hurst, Sofia Dias

**Affiliations:** Centre for Reviews and Dissemination, University of York, York, YO10 5DD UK

**Keywords:** Heterogeneity, Indirect comparison, Network meta-analysis, Single technology appraisal, Psoriasis

## Abstract

**Background:**

Network meta-analyses (NMAs) of psoriasis treatments, undertaken as part of the NICE Single Technology Appraisal (STA) process, have included heterogeneous studies. When there is inconsistency or heterogeneity across the different comparisons or trials within the network of studies, the results of the NMA may not be valid. We explored the impact of including studies with heterogeneous patient characteristics on the results of NMAs of psoriasis treatments.

**Methods:**

All NMAs undertaken for psoriasis STAs were identified and the included studies tabulated, including patient characteristics that may influence relative treatment effects. In addition to the original network of all studies using licensed treatment doses, a range of smaller, less heterogeneous networks were mapped: ‘no previous biologic use’ (< 25% patients had prior biologic therapy exposure), ‘Psoriasis Area and Severity Index score ≤ 25’, ‘weight ≤ 90 kg’ and ‘white ethnicity’ (≥ 90% patients were white).

**Results:**

Sixty-nine studies were included in our synthesis (34,924 participants). A random effects model with a log-normal prior distribution was chosen for each of the subgroup NMAs. Heterogeneity was reduced for the four smaller networks. There were no significant differences in the relative treatment effect (PASI 75 response) for each treatment across the five NMAs, with all credible intervals overlapping, although there were noticeable differences. Treatment rankings based on the median relative risks were also generally consistent across the networks. However, the NMA that included only studies in which < 25% patients had prior biologic therapy exposure had slightly different treatment rankings; the anti-TNF therapies certolizumab pegol and infliximab ranked higher in this network than any other network, although credible intervals were large.

**Conclusions:**

This work has highlighted potential differences in treatment response for biologic-naïve patients. When conducting NMAs in any area, heterogeneity in patient characteristics of included trials should be carefully assessed and effect modification related to certain patient characteristics investigated through clinically relevant subgroup analyses.

## Background

Network meta-analysis (NMA) has become increasingly popular over recent years for estimating the relative effectiveness of several treatments in the absence of direct head-to-head evidence. When direct and indirect evidence is combined in a meta-analysis, there is a risk that patients in different trials differ in terms of demographics, disease, or other patient characteristics. There can also be differences in trial specific features, such as country of origin and trial design. If these differences are effect modifiers, they can result in between-study heterogeneity and create biased comparisons. In a NMA context, such biases and heterogeneity can also lead to inconsistency, i.e. conflict between direct and indirect evidence on the same comparison. It is therefore important to adjust for effect modifiers in a NMA; this can be done by restricting inclusion in the NMA to certain subgroups of patients with similar characteristics or by conducting meta-regression. Focusing the inclusion criteria on key participant or study characteristics to produce smaller, more homogenous networks can reduce the risk of both heterogeneity and inconsistency, and give more valid results [1]. Alternatively, meta-regression, on for example the average weight or proportion of included patients with certain characteristics, can also be conducted. When conducting network meta-regression, a sufficient number of studies is needed to estimate independent coefficients for each treatment comparison. Otherwise, additional assumptions of common regression coefficients must be made, which may not be clinically plausible. In addition, results are often uncertain and hard to interpret. Therefore, it is often more useful to identify clinically meaningful discrete participant and study characteristics which could be expected to lead to different decisions, and restrict inclusion in the NMA.

Previous work carried out for the National Institute for Health and Care Excellence (NICE) has highlighted that several NMAs undertaken for NICE single technology appraisals (STAs) of psoriasis treatments have included heterogeneous studies. However, the very short timeframe of a STA does not allow sufficient time to fully explore the impact of heterogeneity on the NMA results [2]. Therefore, this small methodological project aimed to explore the impact of heterogeneous patient characteristics on the results of a NMA, using data from NICE STAs of psoriasis treatments, since we identified this as an area where previous NMAs have included studies with heterogeneous patient characteristics.

There have been several NICE STAs of systemic therapies for the second-line treatment of moderate-to-severe plaque psoriasis. Psoriasis is a chronic, inflammatory immune-mediated skin disorder with a prevalence of around 3% in the UK [3]. Standard first-line treatment includes topical therapy, or systemic non-biologic therapies or phototherapy for patients with more severe disease. For adults with moderate-to-severe psoriasis who do not respond to, are intolerant of, or have a contraindication to standard systemic therapies and phototherapy, NICE recommends systemic biologic therapies, apremilast or dimethyl fumarate.

The severity of psoriasis is measured using the Psoriasis Area and Severity Index (PASI), which combines the assessment of severity of lesions and the area affected into a single score. PASI is also used to assess response to psoriasis treatment, presented as a percentage response rate; PASI 75 response is a 75% or greater improvement in PASI score, PASI 90 response is a 90% or greater improvement and PASI 100 response is 100% improvement in PASI score (total skin clearance).

The key objectives of this methodological project were:

1. To identify NMAs undertaken as part of a STA of a second-line therapy for moderate -to -severe plaque psoriasis.
2. To identify and tabulate all relevant studies included in the NMAs, recording patient and study characteristics that may influence relative treatment effects (PASI response).
3. To map a range of smaller, less heterogeneous networks.
4. To run the NMAs and compare results with the results of the overall network of evidence.

## Methods

Two researchers (RW and SS) independently screened the NICE website for STAs of second-line therapies for moderate -to- severe plaque psoriasis that included a NMA. The researchers also identified any sensitivity analyses undertaken by the company who undertook the NMA, as an indication of the characteristics that may be considered to have an impact on relative treatment effectiveness.

All studies included in the NMAs were tabulated. Additional randomised controlled trials (RCTs) of second-line therapies for psoriasis were not sought since the search strategies used in the STAs were adequate and the aim of this methodological project was to compare results of NMA subgroups with the original network, rather than to update the previous NMAs. Details of important patient and study characteristics that may influence relative treatment effects were tabulated, such as timeframe at which treatment response was assessed, drug dose, concomitant psoriatic arthritis and prior treatments received (i.e. biologic naïve versus biologic experienced patients). Dermatologists who had acted as clinical advisors to the Centre for Reviews and Dissemination/Centre for Health Economics Technology Assessment Group in previous STAs of second-line therapies for psoriasis were emailed regarding their opinion on the characteristics considered most likely to have an impact on the relative effectiveness of psoriasis treatments on PASI response. The outcome used in the analysis was PASI 75 response, as it is the most widely reported response outcome in the included trials and is used as a measure of treatment response in clinical practice.

Study details were obtained from tables presented as part of the STA of brodalumab [4], supplemented with data presented in primary study reports, where necessary. The brodalumab appraisal was chosen as the primary source of data because it included comprehensive study characteristics tables. The tables were independently checked for accuracy and completeness by a second researcher using tables from two different STAs, supplemented with data presented in primary study reports. All missing data/discrepancies were added/corrected using the original study reports.

Study and patient characteristics considered most likely to have an impact on relative treatment effectiveness were compared for each of the primary studies. New networks, including only studies with similar study and patient characteristics, were defined and mapped using the netmeta package [5] in R [6]. This package uses contrast-level data to create plots of all the trials included in the NMA, highlighting the number of trials between each treatment. All networks were checked for connectivity, making sure that all interventions were directly connected to at least one other intervention, forming one linked network.

Binomial logit-link models were used for the NMAs [2]. Both fixed effect and random effects models were fitted for each network. The choice of prior distributions for the between-study variance was also explored. Model fit was assessed by comparing the total residual deviance to the number of data points in the model. Models were compared using the deviance information criterion (DIC) which accounts for model fit and complexity. The model with a lower DIC (a difference in value of 3 is seen as meaningful) was selected. Where the DIC were within 3 points of each other, the simplest model with fewer parameters was chosen.

## Results

### Review of NICE technology appraisals

There have been ten NICE STAs of systemic therapies for the second-line treatment of moderate-to-severe plaque psoriasis. The second-line systemic therapies that have been appraised are the anti-tumour necrosis factor (TNF) alpha therapies adalimumab, infliximab and certolizumab pegol; the anti-interleukin (IL)-12/23 ustekinumab; the anti-IL-17 therapies secukinumab, ixekizumab and brodalumab; the anti-IL-23 tildrakizumab; the anti-phosphodiesterase (PDE) 4 apremilast; and the nuclear factor (erythroid-derived 2)-like 2 (Nrf2) activator dimethyl fumarate. Other than infliximab, which is only recommended for patients with very severe disease, each of the company submissions included a NMA (see Table 1).

**Table 1:** NICE single technology appraisals of systemic therapies for psoriasis that include network meta-analyses

Psoriasis systemic therapy	Treatment class	Number of trials included in NMA	Sensitivity analyses undertaken
Adalimumab (TA146, 2008) [7]	Anti-TNF-alpha	18 randomised controlled trials (RCTs)	N/A
Ustekinumab (TA180, 2009) [8]	Anti-IL-12/23	20 RCTs	N/A
Secukinumab (TA350, 2015) [9]	Anti-IL-17	26 RCTs	Baseline PASI score; psoriasis duration; prior biologic therapy exposure; baseline Dermatology Life Quality Index (DLQI) score
Apremilast (TA419, 2016) [10]	Anti-PDE4	22 RCTs	Prior biologic therapy exposure
Ixekizumab (TA442, 2017) [11]	Anti-IL-17	40 RCTs	All treatment doses (base case included only NICE-approved doses)
Dimethyl fumarate (TA475, 2017) [12]	Nrf2 activator	37 RCTs	N/A
Brodalumab (TA511, 2018) [4]	Anti-IL-17	59 RCTs	NICE-approved treatment doses; timing of primary outcome assessment; trial size; prior biologic therapy exposure; baseline PASI score
Certolizumab pegol (TA574, 2019) [13]	Anti-TNF-alpha	65 RCTs	Prior biologic therapy exposure
Tildrakizumab (TA575, 2019) [14]	Anti-IL-23	45 RCTs	Timing of primary outcome assessment

### Patient characteristics that may contribute to heterogeneity in relative treatment effects

Sensitivity analyses undertaken alongside the STA NMAs related to the following study/patient characteristics: size of the trial; licensed and NICE approved treatment doses; timing of primary outcome assessment; patients’ baseline PASI score; patients’ baseline DLQI score; duration of disease; and prior exposure to biologic therapy. Two dermatologists (Professor Catherine Smith and Dr Phil Hampton) provided advice on the study and patient characteristics considered most likely to have an impact on the relative effectiveness of psoriasis treatments on PASI response. Important characteristics for which adequate data were available in the studies of psoriasis treatments were patient weight, exposure to previous biologic therapy, white versus non-white ethnicity and baseline PASI score.

### Network identification

We identified 72 studies from previous NMAs of STAs of second-line therapies for moderate to severe plaque psoriasis. We excluded any studies with unlicensed treatments or treatment doses, of which there were two. One study was excluded due to the results being unpublished. Therefore, we included 69 studies in our synthesis (34,924 participants). Characteristics of patients included in the 69 RCTs included in the networks are presented in Additional file 1.

**Fig. 1:**
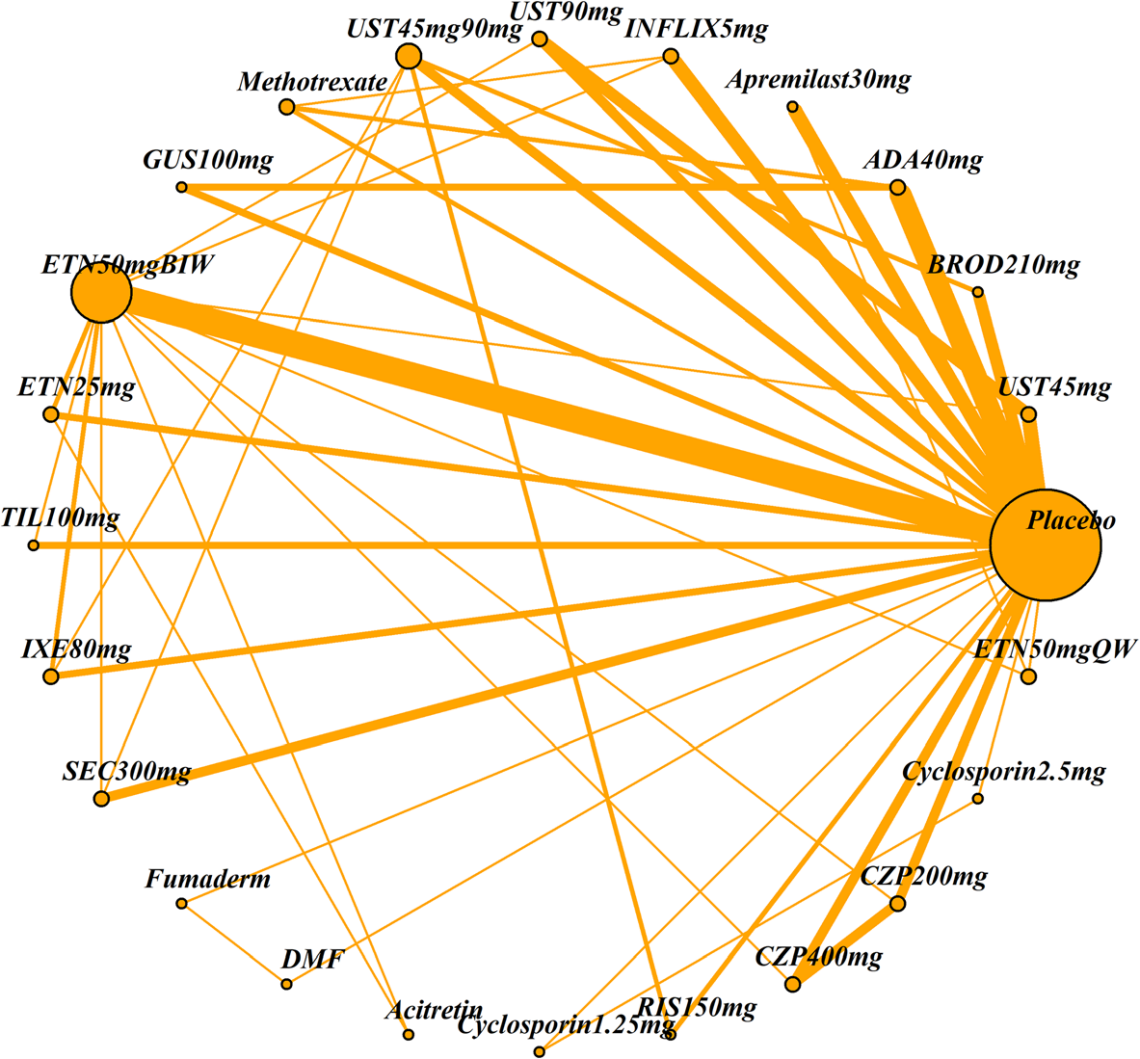
Network of all studies with licensed treatment doses

**Fig. 2:**
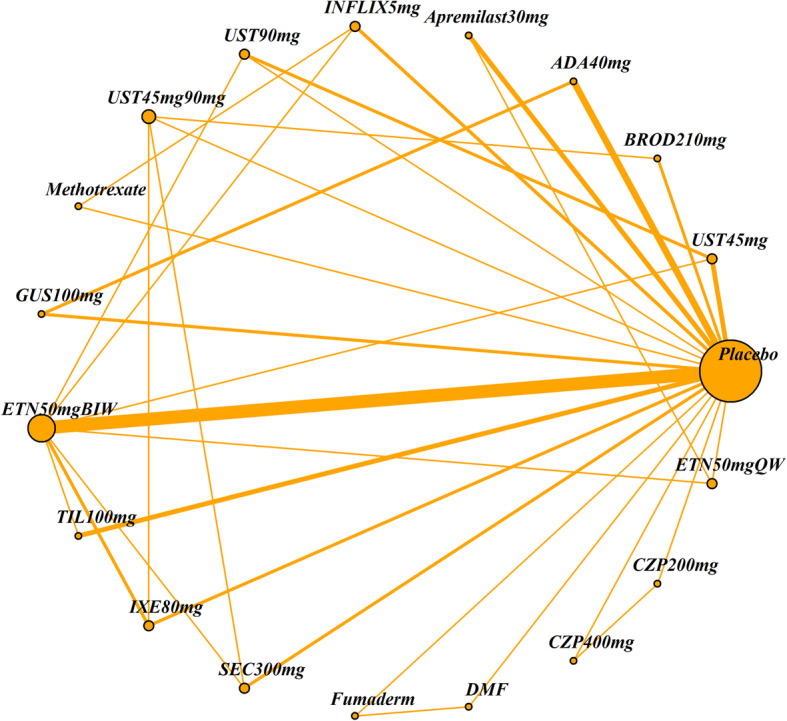
Network of studies of patients with no previous biologic exposure (< 25%)

**Fig. 3:**
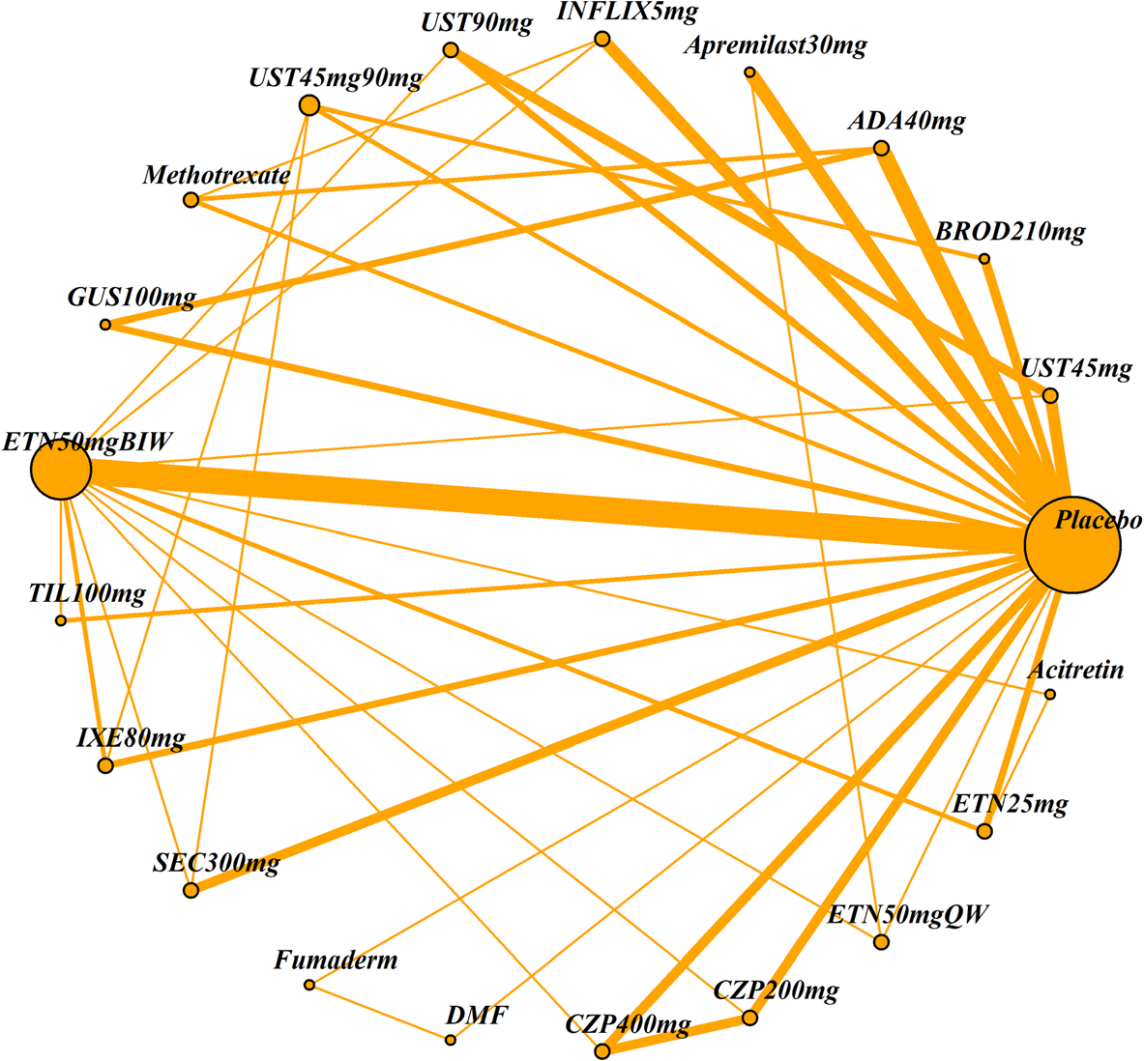
Network of studies of patients with PASI < 25%

**Fig. 4:**
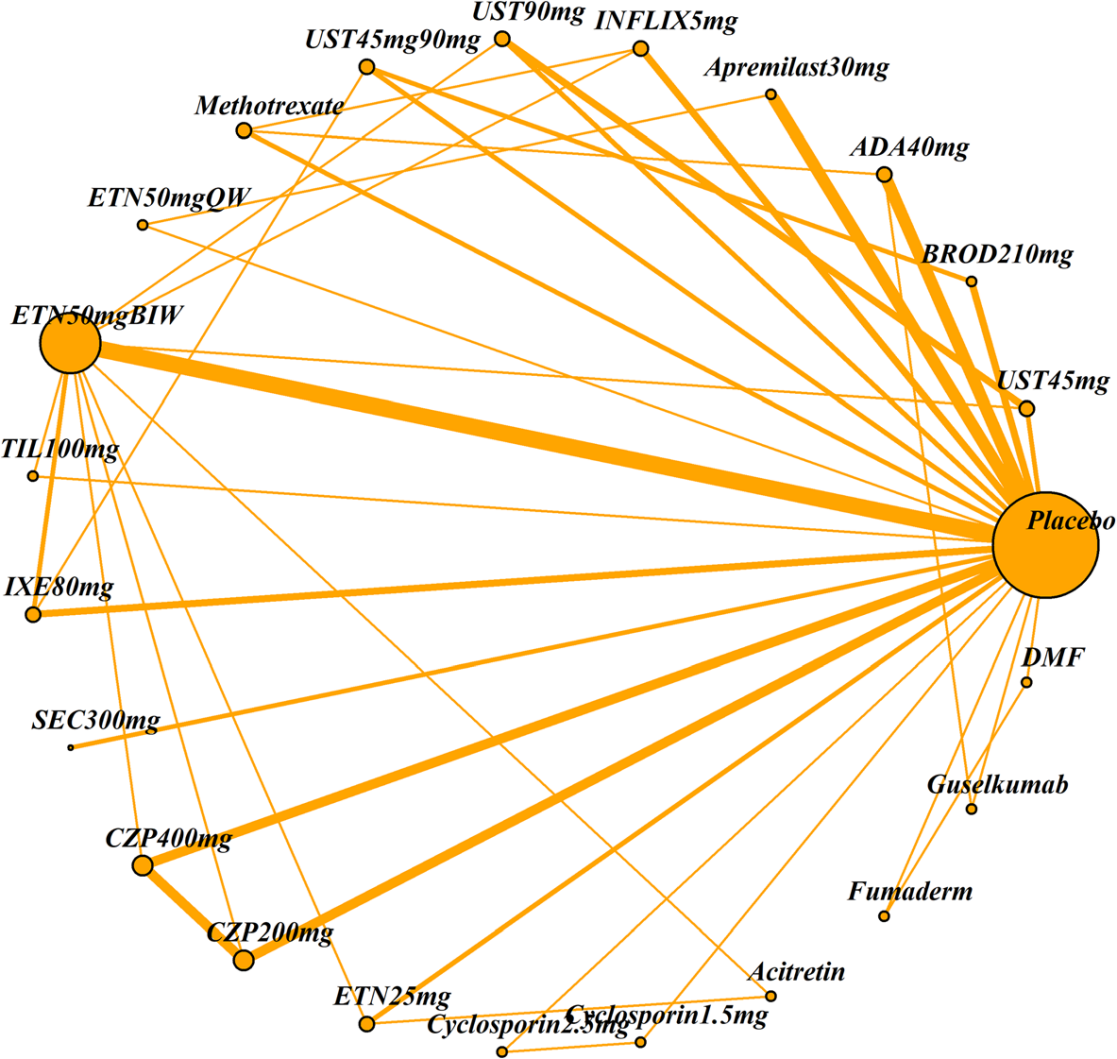
Network of studies of patients with weight ≤ 90 kg

**Fig. 5:**
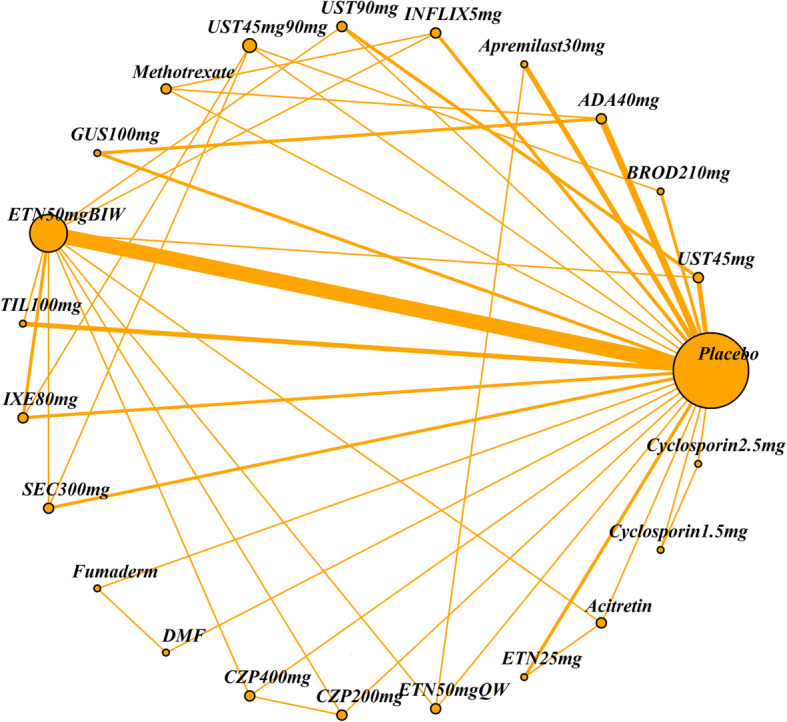
Network of studies including > 90% white patients

The impact of four patient characteristics on relative treatment effectiveness was investigated by producing four smaller networks: ‘no previous biologic use’ (< 25% patients had prior exposure to a biologic therapy), ‘PASI ≤ 25’ (average PASI score was 25 or less), ‘weight ≤ 90 kg’ (average weight was 90 kg or less) and ‘white ethnicity’ (≥ 90% patients were white). Cut-off choice was informed by clinical opinion as well as being pragmatically chosen in order to ensure a sufficient number of studies was still included in each network. The studies included in each of the four networks and the original (all licensed doses) network are listed in Table 2. The network diagrams are shown in Figs. 1, 2, 3, 4 and 5. The width of the connecting lines is proportional to the number of trial level comparisons available and the size of the nodes is proportional to the number of patients who received the corresponding treatment.

**Table 2:** Studies included in each network meta-analysis

Studies	All licensed doses (*N* = 69)	Patients with no previous biologic use (< 25% had previous use) (*N* = 34)	Patients with PASI score ≤ 25 (*N* = 59)	Patients with weight ≤ 90 kg (*N* = 28)	White patients (≥ 90% white) (*N* = 42)
AMAGINE1 2016	✓		✓		✓
AMAGINE2 2015	✓		✓		✓
AMAGINE3 2015	✓	✓	✓	✓	✓
Nakagawa 2016	✓	✓		✓	
Papp 2012	✓		✓	✓	
CHAMPION 2008	✓		✓	✓	✓
Goldminz 2015	✓		✓		
Cai 2016	✓	✓		✓	
REVEAL 2008	✓	✓	✓		✓
Asahina 2010	✓			✓	
Gordon 2006	✓		✓		✓
XPLORE 2015	✓		✓		✓
Bissonnette 2013	✓		✓		✓
VOYAGE1 2017	✓	✓	✓		
VOYAGE2 2017	✓	✓	✓		
PSOR005 2012	✓		✓		✓
ESTEEM1 2015	✓		✓		✓
ESTEEM2 2015	✓		✓		✓
Ohtsuki 2017	✓	✓	✓	✓	
LIBERATE 2016	✓	✓	✓	✓	✓
Leonardi 2003	✓		✓		
Gottlieb 2003	✓		✓		✓
Papp 2005	✓		✓		✓
VandeKerkhof 2008	✓		✓	✓	✓
Bagel 2012	✓	✓	✓		
Bachelez 2015	✓	✓	✓	✓	
Tyring 2006	✓		✓		
PRISTINE 2013	✓	✓	✓	✓	
M10114 2011	✓	✓	✓		✓
M10315 2011	✓	✓	✓		✓
reSURFACE2	✓	✓	✓	✓	✓
PIECE 2016	✓	✓	✓		✓
Yang 2012	✓		✓	✓	
EXPRESS 2005	✓		✓	✓	
Chaudhari 2001	✓	✓	✓	✓	✓
SPIRIT 2004	✓		✓		✓
EXPRESSII 2007	✓	✓	✓		✓
Torii 2010	✓			✓	
RESTORE1 2011	✓	✓	✓	✓	✓
UNCOVER1 2016	✓		✓		✓
UNCOVER2 2015	✓	✓	✓		✓
UNCOVER3 2015	✓	✓	✓		✓
IXORAS 2017	✓	✓	✓	✓	✓
FEATURE 2015	✓		✓		✓
ERASURE 2014	✓		✓	✓	
FIXTURE 2014	✓	✓	✓	✓	
JUNCTURE 2015	✓	✓	✓		✓
CLEAR 2015	✓	✓	✓	✓	
PEARL 2011	✓	✓	✓	✓	
PHOENIX1 2008	✓		✓		✓
PHOENIX2 2008	✓		✓		✓
LOTUS 2013	✓	✓	✓	✓	
ACCEPT 2010	✓	✓	✓		✓
Igarashi 2012	✓	✓		✓	
BRIDGE 2017	✓	✓	✓		✓
Caproni 2009	✓		✓		✓
Gisondi 2008	✓		✓		✓
Meffert	✓				✓
PappD 2015	✓	✓			
ReSURFACE1	✓	✓	✓	✓	
ultIMMA1	✓				
ultIMMA2	✓				
METOP	✓	✓	✓		✓
Krueger	✓		✓		
Reich 2012	✓	✓	✓	✓	✓
CIMPACT 2018	✓		✓	✓	✓
CIMPASI1 2018	✓		✓		✓
CIMPASI2 2018	✓		✓		✓
UNVEIL	✓	✓	✓	✓	✓

### Model fit

In all models, both a uniform (0, 3) prior distribution and an empirically based log normal (− 2.70, 1.52^2^) informative prior distribution [15] were used. The random effects model with a uniform prior distribution was found to have a superior fit for the network of all studies with licensed doses (Table 3) as the residual deviance was closer to the number of unconstrained data points than the fixed effects model and the random effects model with log-normal prior distribution. The deviance information criterion (DIC) was also lower for the uniform prior random effects model than the other two models.

**Table 3:** Measures of goodness of fit of fixed and random effects models for each of the five network meta-analyses

Measure of goodness of fit	Random effects (uniform prior)	Random effects (log-normal prior)	Fixed effects
Licensed doses network			
Residual deviance^a^	162.78	177.54	209.77
pD	117.98	106.29	91.61
Deviance information criterion (DIC)	280.76	283.83	301.38
Between-study standard deviation, posterior median (95% credible interval)	0.31 (0.17–0.45)	0.19 (0.12–0.28)	–
Network of patients with no previous biologic use (< 25% had previous use)			
Residual deviance^b^	82.10	82.88	88.85
pD	59.12	56.3	52.45
Deviance information criterion (DIC)	141.22	139.20	141.30
Between-study standard deviation, posterior median (95% credible interval)	0.19 (0.01–0.41)	0.14 (0.09–0.23)	–
Network of patients with PASI score ≤ 25			
Residual deviance^c^	143.89	152.67	173.06
pD	99.16	90.58	79.61
Deviance information criterion (DIC)	243.05	243.26	252.67
Between-study standard deviation, posterior median (95% credible interval)	0.2574 (0.114–0.408)	0.16 (0.10–0.24)	–
Network of patients with weight ≤ 90 kg			
Residual deviance^d^	66.40	74.17	80.02
pD	51.59	44.78	42.14
Deviance information criterion (DIC)	117.99	118.95	122.16
Between-study standard deviation, posterior median (95% credible interval)	0.40 (0.08–0.76)	0.15 (0.09–0.24)	**–**
Network of ≥ 90% white patients			
Residual deviance^e^	100.57	112.47	126.65
pD	78.57	71.62	63.83
Deviance information criterion (DIC)	179.14	184.09	190.48
Between-study standard deviation, posterior median (95% credible interval)	0.311 (0.13–0.51)	0.17 (0.10–0.25)	–

^a^165 unconstrained data points, pD number of parameters for licensed doses network

^b^80 unconstrained data points, pD number of parameters

^c^143 unconstrained data points, pD number of parameters

^d^65 unconstrained data points, pD number of parameters

^e^103 unconstrained data points, pD number of parameters

The random effects model with a log-normal prior distribution was chosen for the network of patients with no previous biologic use (< 25% patients had previous biologic use), the network of patients with PASI score ≤ 25, the network of patients with weight ≤ 90 kg and the network of ≥ 90% white patients (Table 3). The DIC and residual deviance was much lower for the random effects models than the fixed effects models. Although the DIC was very similar between the random effects models, the log-normal prior model was chosen as it had a much smaller number of parameters (pD) than the uniform prior model.

### Heterogeneity

**Fig. 6:**
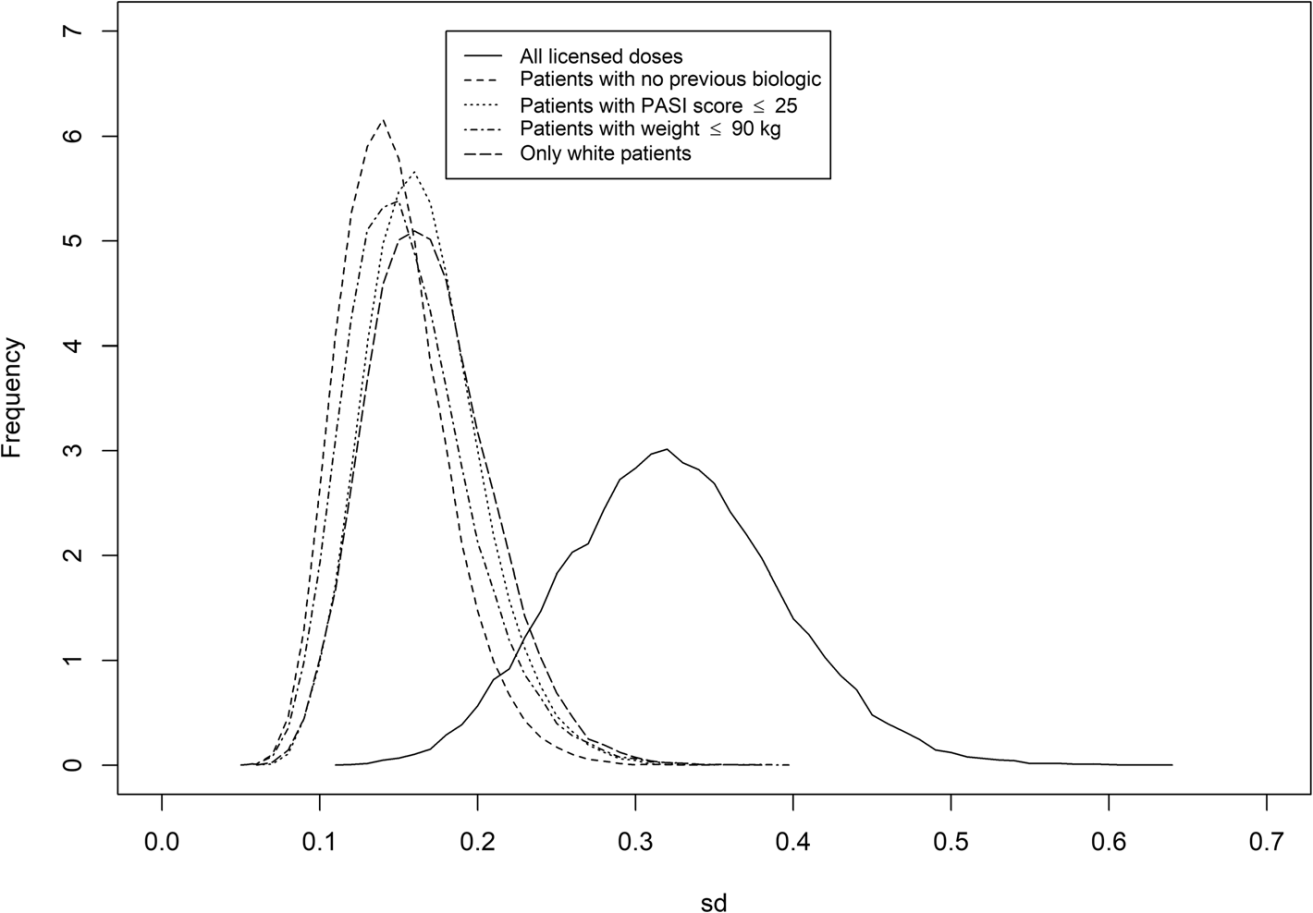
Posterior between-study heterogeneity density for the five NMAs

The network of all studies with licensed doses had the highest between-study heterogeneity (0.31, 95% CrI 0.17–0.45). The between-study heterogeneity was reduced for the four smaller networks, which all had similar values. However, the network of patients with no previous biologic use had the smallest heterogeneity (0.14, 95% CrI 0.09–0.23), alongside the network of patients with weight ≤ 90 kg (0.15, 95% CrI 0.09–0.24). The densities of the posterior between-study heterogeneity for each network meta-analysis are shown in Fig. 6.

### Effects of the interventions

Relative risk ratios for each treatment compared against placebo are shown in Table 4. Across the five NMAs, the relative risks for each treatment appear to be similar, with all credible intervals overlapping. However, there are some noticeable differences. Etanercept 50 mg (once-weekly) had a higher relative treatment effect of achieving PASI 75 in the licensed doses network (10.67, 95% CrI 7.96–13.53) compared to all other networks and methotrexate had a higher relative effect in the network of patients with no previous biologic use (< 25% had previous use) (10.47, 95% CrI 6.73–14.41) compared to the other networks. In the ≥ 90% white patients network, secukinumab had a higher relative treatment effect than in all other networks (18.67, 95% CrI 16.22–20.81) and guselkumab had a lower relative treatment effect compared to all the other networks (15.30, 95% CrI 10.89-18.39). However, their credible intervals were large.

**Table 4:** Median risk ratio for each treatment compared against placebo in all five network meta-analyses

Treatment	Median risk ratio versus placebo–PASI 75 (95% CrI)				
	All licensed doses	No previous biologic use (< 25%)	PASI score ≤ 25	Weight ≤ 90 kg	≥ 90% white patients
Adalimumab 40 mg	12.74 (11.00–14.49)	12.48 (10.91-14.13)	13.09 (11.72-14.57)	12.87 (10.29-15.39)	13.18 (11.21-15.15)
Brodalumab 210 mg	16.76 (15.12–18.53)	16.45 (14.66–18.31)	16.62 (15.20–18.18)	16.73 (14.97–18.58)	16.56 (15.02–18.24)
Certolizumab 200 mg	12.07 (9.62–14.54)	13.93 (8.63–18.20)	12.08 (10.30–13.94)	11.71 (9.11–14.27)	12.13 (10.02–14.26)
Certolizumab 400 mg	13.47 (11.09–15.80)	15.73 (10.81–19.08)	13.42 (11.65–15.23)	13.04 (10.53–15.46)	13.48 (11.42–15.52)
Etanercept 25 mg	7.61 (5.52–10.11)	–	7.64 (6.20–9.20)	–	7.89 (5.60–10.51)
Etanercept 50 mg once–weekly	10.67 (7.96–13.53)	5.08 (3.50–7.07)	6.16 (4.69–7.90)	5.57 (3.91–7.65)	7.07 (4.57–10.20)
Etanercept 50 mg twice per week	9.90 (8.68–11.21)	9.46 (8.27–10.77)	10.40 (9.47–11.40)	9.85 (8.27–11.55)	10.33 (9.01–11.75)
Guselkumab 100 mg	17.06 (15.30–18.91)	16.68 (15.07–18.42)	16.83 (15.32–18.46)	–	15.30 (10.89–18.39)
Infliximab 5 mg	16.22 (14.37–18.15)	16.88 (14.66–19.03)	15.46 (13.85–17.17)	14.19 (11.76–16.56)	15.38 (13.41–17.36)
Ixekizumab 80 mg	17.64 (16.06–19.36)	17.42 (15.89–19.09)	17.79 (16.30–19.41)	17.16 (14.67–19.33)	17.75 (16.23–19.41)
Risankizumab 150 mg	16.46 (14.37–18.47)	–	–	–	–
Secukinumab 300 mg	16.45 (14.79–18.23)	16.03 (14.41–17.73)	16.43 (15.03–17.96)	16.12 (14.55–17.82)	18.67 (16.22–20.81)
Ustekinumab 45 mg	13.59 (11.79–15.44)	12.20 (10.32–14.14)	13.46 (12.17–14.84)	13.04 (10.35–15.63)	13.68 (11.95–15.48)
Ustekinumab 90 mg	14.67 (12.85–16.54)	13.36 (11.29–15.38)	14.51 (13.20–15.95)	14.36 (10.18–17.57)	14.64 (13.00–16.37)
Ustekinumab (45 mg or 90 mg)	12.85 (11.07–14.67)	12.96 (11.05–14.94)	13.19 (11.79–14.66)	13.11 (10.99–15.20)	13.14 (11.35–14.95)
Tildrakizumab 100 mg	14.86 (12.49–17.02)	15.03 (13.18–16.91)	15.82 (14.25–17.50)	15.28 (13.31–17.21)	16.21 (14.20–18.16)
Apremilast	5.80 (4.20–7.61)	3.77 (2.48–5.59)	5.17 (4.01–6.61)	3.91 (2.60–5.79)	5.46 (4.12–7.10)
Dimethyl Fumarate	2.97 (1.44–5.73)	2.97 (1.77–4.88)	2.97 (1.71–5.01)	–	2.96 (1.71–4.98)
Fumaderm	3.31 (1.62–6.26)	3.32 (1.97–5.41)	3.31 (1.94–5.53)	–	3.30 (1.92–5.48)
Methotrexate	6.15 (4.07–8.65)	10.47 (6.73–14.41)	6.50 (4.69–8.60)	5.49 (3.56–8.12)	6.30 (4.37–8.61)
Acitretin	4.024 (1.55–8.39)	–	4.07 (1.59–8.29)	–	4.29 (1.74–8.43)
Cyclosporin 1.5 mg	8.10 (2.41–16.91)	–	–	–	2.14 (0.38–10.53)
Cyclosporin 2.5 mg	7.10 (2.02–16.34)	–	–	–	6.76 (2.07–16.03)

**Fig. 7:**
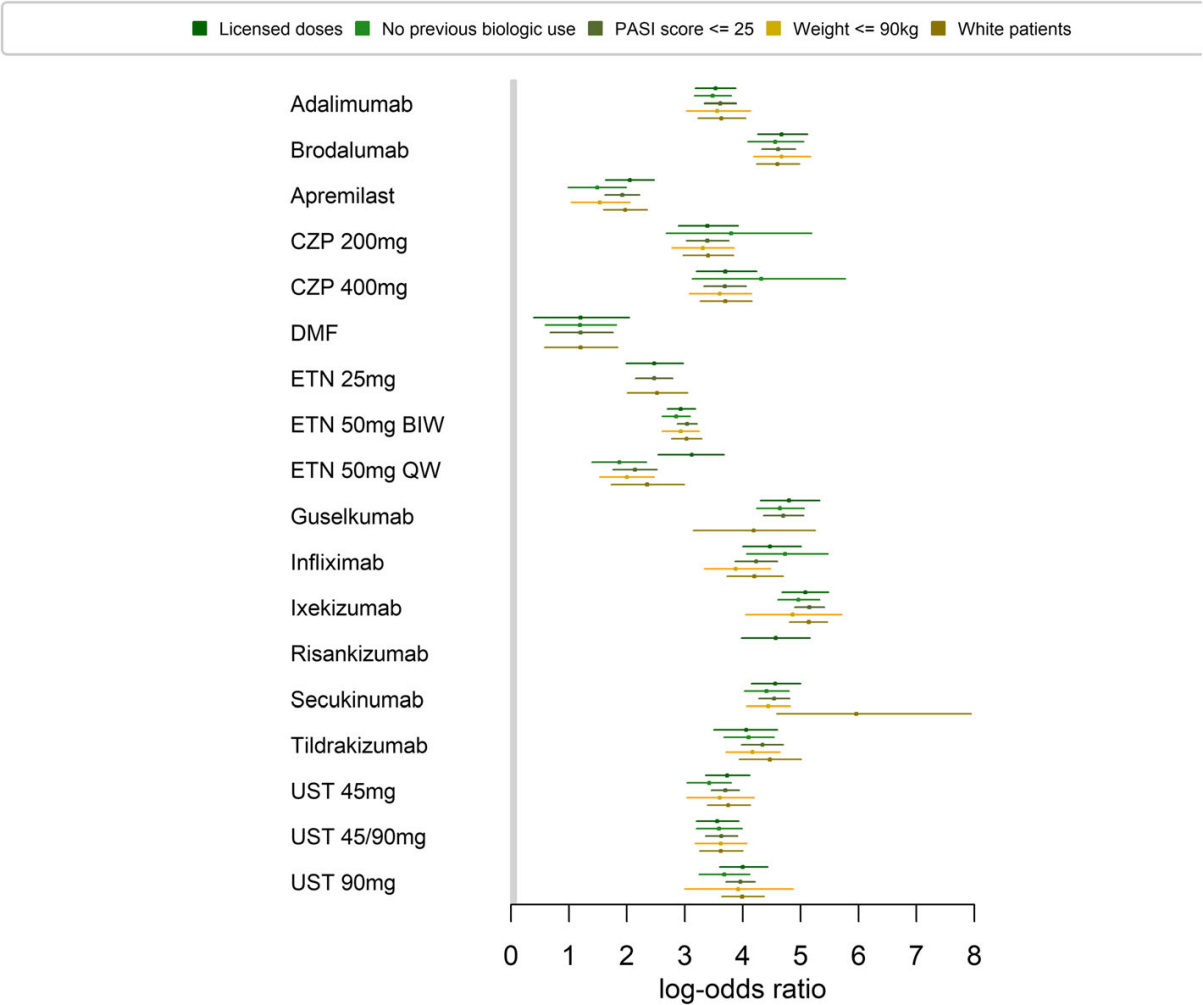
Relative treatment effects split by network group for each treatment

Log-odds ratios for each network and for each treatment compared to placebo are shown in Fig. 7. Absolute probabilities of achieving PASI 75 for each treatment across the five networks are shown in Additional file 2.

The median rankings of treatments based on the relative risks are shown in Table 5. Ixekizumab ranks best in all networks, except the network with predominantly white patients, in which secukinumab ranks best. Dimethyl fumarate ranks worst in all five networks. The rankings are generally consistent across the networks. However, the NMA that included only studies in which less than 25% of patients had prior exposure to a biologic therapy had slightly different treatment rankings; the anti-TNF therapies certolizumab pegol (median rank of 8 [95% CrI 2–13] for the 200 mg dose and 6 [95% CrI 1–11] for the 400 mg dose) and infliximab (median rank of 3 [95% CrI 3–11]) ranked higher in this network group than any of the other networks, indicating that these two therapies may work better in patients who have not previously received biologic therapy, although we note the large uncertainty in these rankings. However, biologic experienced patients are more likely to have had prior exposure to an anti-TNF therapy (i.e. adalimumab or etanercept) which may explain why subsequent response to the anti-TNF therapies certolizumab pegol and infliximab was lower in the networks that did not include primarily biologic-naïve patients.

**Table 5:** Median rank of treatments according to PASI 75 response in each of the five networks

Treatment	Median rank (95% CrI)				
	Licensed doses *N* = 69	No previous biologic use (< 25%) *N* = 34	PASI score ≤ 25 *N* = 59	Weight ≤ 90 kg *N* = 27	≥ 90% white patients *N* = 42
Adalimumab	11 (8–14)	11 (8–12)	10 (7–12)	9 (5–11)	10 (7–12)
Apremilast	17 (16–18)	15 (14–16)	16 (15–16)	14 (13–14)	16 (15–16)
Brodalumab	3 (1–6)	4 (1–7)	3 (2–5)	2 (1–4)	4 (2–6)
Certolizumab 200 mg	13 (9–15)	8 (2–13)	12 (9–12)	11 (7–12)	12 (8–13)
Certolizumab 400 mg	10 (7–13)	6 (1–11)	9 (7–11)	8 (5–11)	9 (6–11)
DMF	18 (17–18)	16 (14–16)	17 (17–17)	–	17 (17–17)
Etanercept 25 mg	16 (15–17)	–	14 (14–15)	–	14 (13–16)
Etanercept 50 mg (twice per week)	15 (14–15)	13 (12–13)	13 (13–13)	12 (11–12)	13 (12–14)
Etanercept 50 mg (once-weekly)	14 (10–16)	14 (14–15)	15 (14–16)	13 (13–14)	15 (13–16)
Guselkumab	2 (1–6)	3 (1–7)	2 (2–5)	–	6 (2–12)
Infliximab	5 (2–8)	3 (1–7)	6 (3–7)	6 (3–10)	5 (3–9)
Ixekizumab	1 (1–4)	1 (1–4)	1 (1–1)	1 (1–5)	2 (1–3)
Risankizumab	4 (1–8)	–	–	–	–
Secukinumab	4 (2–7)	5 (2–7)	4 (2–6)	3 (1–5)	1 (1–3)
Tildrakizumab	7 (4–12)	7 (4–9)	5 (3–7)	4 (2–7)	4 (3–7)
Ustekinumab 45 mg	10 (8–13)	11 (8–12)	9 (8–12)	8 (5–11)	9 (6–12)
Ustekinumab 45 mg/90 mg	11 (8–14)	10 (7–12)	10 (7–12)	8 (5–11)	10 (6–12)
Ustekinumab 90 mg	8 (5–10)	9 (7–12)	7 (6–8)	6 (1–11)	7 (5–9)
Total number of treatments	18	16	17	14	17

The network of primarily white patients also had slightly different treatment rankings; secukinumab ranked higher and guselkumab ranked lower than in the other networks, although there was large uncertainty for the guselkumab result. Data on ethnicity was often not reported in the included studies, so some assumptions had to be made based on the location of the study when extracting data from primary studies, adding further uncertainty to the results for this network.

### Sensitivity analysis

Some studies of the earlier treatments for psoriasis, adalimumab, etanercept and infliximab, did not report prior biologic use; however, they may have had largely biologic-naïve patient populations as biologics were not widely available at the time they were conducted. Therefore, all the studies not already included in the network of patients who had no prior biologic exposure (< 25% patients) were screened and studies conducted prior to 2007, where prior biologic use was not reported, were added to the network. The cut-off of 2007 was chosen to ensure that all the earliest studies were included. Six studies conducted prior to 2007 were identified and included in the network: Gottlieb et al. (2003) [16], Leonardi et al. (2003) [17], Papp et al. (2005) [18], Reich et al. (2005) [19], Gordon et al. (2006) [20] and Tyring et al. (2006) [21]. The random effects model with a log-normal prior distribution was chosen for the network of patients with no previous biologic use (< 25% patients had previous biologic use) (see Additional file 3, Table 1).

The results from the sensitivity analysis were very similar to the main results (see Additional file 3, Table 2). There were minimal changes to the risk ratios, with very little difference in the anti-TNF drugs adalimumab, infliximab and etanercept. There were a few small changes to other treatments. The median ranking of guselkumab changed from 3 to 4, with the same credible interval of 1–7. The median ranking of apremilast and DMF dropped one rank each, with the addition of etanercept 25 mg to the network, making the total number of treatments 17, rather than 16.

## Discussion

The smaller networks investigated were less heterogeneous, with between-study standard deviation ranging from 0.14 (95% CrI 0.09–0.23) for the network of patients with no previous biologic use to 0.17 (95% CrI 0.10–0.25) for the network of predominantly white patients, in comparison with the network of all studies with licensed doses (0.31, 95% CrI 0.17–0.45). The reduction in heterogeneity in the network of patients with no previous biologic use could be due to the population being more clinically homogenous. Previous biologic use may be an important effect modifier and so excluding patients with previous biologic use may have removed a significant source of heterogeneity.

Results for most of the NMAs were consistent, in terms of treatment rankings for PASI 75 response. The main exception was the NMA of studies in which ≤ 25% patients had prior exposure to a biologic therapy; in this network, results were better for the anti-TNF therapies certolizumab pegol and infliximab than in the other networks. Whilst this could simply reflect the fact that studies in which a higher proportion of patients had prior exposure to a biologic therapy had used an anti-TNF as the prior therapy (i.e. adalimumab or etanercept), this may be an important effect modifier. Prior biologic therapy exposure was the most commonly conducted sensitivity analysis amongst the NICE STAs of systemic therapies for psoriasis that included a NMA (see Table 1) and our results confirm the importance of considering this as a potential effect modifier.

Meta-regression is another method commonly used to adjust for effect modifiers. However, this requires a sufficient number of studies in order to estimate independent coefficients for each treatment comparison. Additional file 4 presents the number of studies that reported each continuous covariate for each treatment comparison. This shows that there are not enough studies between comparisons to estimate independent coefficients and a common regression coefficient would need to be assumed, which may not be clinically credible. Therefore, analyses were simplified by dichotomising variables according to clinically relevant cut-offs and creating separate networks. Previous work has investigated the effect of baseline risk using meta-regression [22]. Baseline risk is often a proxy for multiple observed and unobserved effect modifiers and does not describe specific individual patient-related treatment effect modifiers. Adjusting for baseline risk in this analysis may not be clinically meaningful for decision making since it is uncertain what determines the baseline risk. Our aim was to characterise heterogeneity based on known and previously hypothesised study-level characteristics that translate to individual patient characteristics, which can be used to focus decision-making on more specific, homogeneous populations.

A limitation of our analysis is the variation in time point at which PASI 75 was assessed in the included studies. In most included studies, the time point for the primary efficacy assessment was week 12, although in some studies it was week 16; adalimumab, apremilast, certolizumab pegol, tildrakizumab and ustekinumab were assessed at week 12 in some studies and week 16 in others. The primary efficacy assessment was week 10 in placebo-controlled trials of infliximab, reflecting the shorter time to treatment effect for this therapy.

Our findings could be investigated further using individual patient data meta-analysis accounting for different important covariates. However, this preliminary approach has highlighted potential differences in treatment response for patients with prior exposure to biologic therapy. Where individual patient data are available, a better characterisation of patients’ prior biologic use could be used to further explore the differences identified.

### Comparison with other results

Treatment rankings for the ‘licensed doses’ NMA were broadly consistent with the results of the NMA undertaken by the guideline development group for the BAD guidelines for biologic therapy for psoriasis, published in April 2017 [23]. The BAD NMA compared ixekizumab, secukinumab, infliximab, ustekinumab, adalimumab, etanercept, methotrexate and placebo. Interventions were ranked in order of efficacy using the surface under the cumulative ranking (SUCRA) curve method. For the outcome, PASI 75 at 3–4 months ixekizumab ranked best (SUCRA 96.4, mean rank 1.3), followed by infliximab (SUCRA 81.2, mean rank 2.3), secukinumab (SUCRA 79.0, mean rank 2.5), ustekinumab (SUCRA 51.9, mean rank 4.4), adalimumab (SUCRA 48.7, mean rank 4.6), etanercept (SUCRA 28.4, mean rank 6.0), methotrexate (SUCRA 14.5, mean rank 7.0) and placebo (SUCRA 0, mean rank 8.0). However, the BAD NMA pooled licensed and unlicensed doses [24]. It included many unlicensed doses that were not included in this analysis as they are not relevant for decision-making. Naïve pooling across doses, without accounting for possible differential dose effects, is not recommended as it can increase heterogeneity due to different treatment definitions. Furthermore, the aim of this analysis was to characterise heterogeneity in networks used by NICE; therefore, only licensed doses were relevant.

A recent article evaluated the association between patient characteristics and response to biologic therapies for psoriasis, using a multicentre longitudinal cohort study; the British Association of Dermatologists Biologic Interventions Register (BADBIR) [25]. This study also found little evidence for predictors of differential treatment response, although only biologic-naïve patients were included in the study.

### Network structure

There was some overlap between networks in terms of included studies (see Table 2). In particular, many of the studies excluded from the ≥ 90% white patients network were included in the network of studies with lighter patients (≤ 90 kg). Only ten studies included patients with a mean weight below 80 kg, nine of which were conducted in Japanese, Chinese or mixed Taiwanese, Chinese and Korean patients (see Additional file 1).

### Recommendations for future research

NMAs of psoriasis treatments undertaken in the future should investigate heterogeneity within the networks and include clinically relevant subgroups to further investigate effect modification related to certain patient characteristics. This recommendation is also appropriate for NMAs in other clinical areas and other fields outside of medicine.

## Conclusions

This work has highlighted potential differences in relative treatment effectiveness for biologic-naïve patients receiving psoriasis treatment. Our results support the assumption that prior exposure to biologic therapy is associated with psoriasis treatment response and confirm the importance of considering this as a potential effect modifier. Future decision-making on psoriasis treatments should consider patients’ prior exposure to biologic therapies.

More broadly, we have demonstrated the importance of assessing heterogeneity in patient characteristics and adjusting for effect modifiers in a NMA, which can be done by restricting inclusion in the NMA to certain subgroups of patients with similar characteristics. Focusing the inclusion criteria to produce smaller, more homogenous networks can reduce the risk of both heterogeneity and inconsistency, and give more valid results.

## Supplementary information

**Additional file 1:.** Characteristics of patients included in the 69 RCTs included in the networks

**Additional file 2:.** Absolute probabilities of achieving PASI 75 across the five networks

**Additional file 3:.** Table 1 Model fit for sensitivity analysis, Table 2 Median risk ratio and median ranks for the sensitivity analysis of patients with no previous biologic use including pre-2007 studies where prior exposure was not reported

**Additional file 4:.** Number of studies that report each continuous covariate for each treatment comparison

## Data Availability

All data generated or analysed during this study are included in this published article (and its supplementary information files).

## Abbreviations

BADBIR: British Association of Dermatologists Biologic Interventions Register
DLQI: Dermatology Life Quality Index
DIC: Deviance information criterion
IL: Interleukin
NICE: National Institute for Health and Care Excellence
NMA: Network meta-analysis
Nrf2: Nuclear factor (erythroid-derived 2)-like 2
PDE: Phosphodiesterase
PASI: Psoriasis Area and Severity Index
RCT: Randomised controlled trial
STA: Single technology appraisal
SUCRA: Surface under the cumulative ranking
TNF: Tumour necrosis factor
