# Supplementary material for: Patient characteristics as effect modifiers for psoriasis biologic treatment response: an assessment using network meta-analysis subgroups
Source: Syst Rev. 2020 Jun 5;9:132. doi: 10.1186/s13643-020-01395-6 (PMC7275463; doi:10.1186/s13643-020-01395-6)
Supplement: Supplementary file 1 — Additional file 1:. Characteristics of patients included in the 69 RCTs included in the networks [file 13643_2020_1395_MOESM1_ESM.docx]

**Additional file 1: Characteristics of patients included in the 69 RCTs included in the networks**

| **Study** | **Intervention / Comparator** | **Primary endpoint timepoint** | **n** | **Age (mean)** | **Male** | **Weight (mean)** | **PASI score**  **(mean)** | **Body surface area affected (mean)** | **Previous biologic** | **Previous non-biologic systemic** | **Psoriatic arthritis** | **Psoriasis years (mean)** | **Ethnicity** | **Country** |
| --- | --- | --- | --- | --- | --- | --- | --- | --- | --- | --- | --- | --- | --- | --- |
| AMAGINE-1, Papp *et al.*, 2016 | Placebo | 12 weeks | 220 | 47 | 73% | 90.4 kg | 19.7 | 26.9% | 46% | 75% | 29% | 21 | 92% white | Europe, Canada and USA |
|  | Brodalumab 140 mg |  | 219 | 46 | 74% | 90.6 kg | 20 | 27.4% | 45% | 65% | 27% | 19 | 90% white | Europe, Canada and USA |
|  | Brodalumab 210mg |  | 222 | 46 | 73% | 91.4 kg | 19.4 | 25.1% | 47% | 70% | 26% | 20 | 91% white | Europe, Canada and USA |
| AMAGINE-2, Lebwohl *et al.*, 2015 | Placebo | 12 weeks | 309 | 44 | 71% | 92 kg | 20.4 | 28% | 29% | 74% | 17% | 18 | 88% white | International |
|  | Brodalumab 140 mg |  | 610 | 45 | 68% | 92 kg | 20.5 | 27% | 29% | 77% | 21% | 19 | 91% white | International |
|  | Brodalumab 210 mg |  | 612 | 45 | 69% | 91 kg | 20.3 | 26% | 29% | 77% | 19% | 19 | 90% white | International |
|  | Ustekinumab (45 mg if <100 kg; 90 mg if >100 kg) |  | 300 | 45 | 68% | 91 kg | 20 | 27% | 28% | 75% | 17% | 19 | 90% white | International |
| AMAGINE-3, Lebwohl *et al.*, 2015 | Placebo | 12 weeks | 315 | 44 | 66% | 89 kg | 20.1 | 28% | 24% | 65% | 19% | 18 | 93% white | International |
|  | Brodalumab 140 mg |  | 629 | 45 | 70% | 89 kg | 20.1 | 29% | 25% | 70% | 21% | 17 | 91% white | International |
|  | Brodalumab 210 mg |  | 624 | 45 | 69% | 90 kg | 20.4 | 28% | 25% | 68% | 20% | 18 | 91% white | International |
|  | Ustekinumab (45 mg if <100 kg; 90 mg if >100 kg) |  | 313 | 45 | 68% | 90 kg | 20.1 | 28% | 24% | 70% | 20% | 18 | 90% white | International |
| Nakagawa *et al.*, 2016 | Placebo | 12 weeks | 38 | 46.6 | 71% | 72.2 kg | 24 | 37.8% | 8% | 89.50% | 18% | 17 | 100% Japanese | Japan |
|  | Brodalumab 70 mg |  | 39 | 43.4 | 87% | 78.2 kg | 27.6 | 39.7% | 13% | 74.40% | 15% | 13 | 100% Japanese | Japan |
|  | Brodalumab 140 mg |  | 37 | 46.4 | 81% | 73.6 kg | 28.5 | 42.7% | 8% | 81.10% | 16% | 15 | 100% Japanese | Japan |
|  | Brodalumab 210 mg |  | 37 | 46.4 | 78% | 72.6 kg | 28 | 43.7% | 14% | 75.70% | 14% | 15 | 100% Japanese | Japan |
| Papp *et al.*, 2012 | Placebo | 12 weeks | 38 | 41.8 | 58% | 86.9 kg | 18.9 | 23.5% | 35% | NR | 18% | 18 | 84% white | International |
|  | Brodalumab 70 mg |  | 39 | 42.1 | 56% | 88.8 kg | 18.8 | 24% | 35% | NR | 21% | 21 | 92% white | International |
|  | Brodalumab 280 mg |  | 42 | 42.3 | 71% | 91.5 kg | 17.9 | 21% | 35% | NR | 19% | 19 | 86% white | International |
|  | Brodalumab 140 mg |  | 39 | 44 | 72% | 92.4 kg | 19.4 | 24.9% | 35% | NR | 28% | 19 | 95% white | International |
|  | Brodalumab 210 mg |  | 40 | 42.1 | 62% | 90.4 kg | 20.6 | 25% | 35% | NR | 30% | 17 | 85% white | International |
| CHAMPION, Saurat *et al.,* 2008 | Placebo | 16 weeks | 53 | 40.7 | 66% | 82.6 kg | 19.2 | 28% | NR | NR | 21% | 19 | 92.5% Caucasian | Europe and Canada |
|  | Adalimumab 40 mg Q2W |  | 108 | 42.9 | 65% | 81.7 kg | 20.2 | 34% | NR | NR | 21% | 18 | 95.4% Caucasian | Europe and Canada |
|  | Methotrexate |  | 110 | 41.6 | 66% | 83.1 kg | 19.4 | 32% | NR | NR | 17% | 19 | 95.5% Caucasian | Europe and Canada |
| Goldminz *et al.*, 2015 | Adalimumab 40 mg Q2W | 16 weeks | 15 | 50.5 | 73% | NR | 16.8 | NR | NR | 40% | 13% | 17 | 80% white | USA |
|  | Methotrexate |  | 15 | 50.3 | 87% | NR | 15.9 | NR | NR | 27% | 20% | 22 | 93% white | USA |
| Cai *et al.*, 2016 | Placebo | 12 weeks | 87 | 43.8 | 67% | 67 kg | 25.6 | 39.3% | 0% | 16% | 12% | 15.8 | 100% Chinese | China |
|  | Adalimumab 40 mg Q2W |  | 338 | 43.1 | 75% | 69.7 kg | 28.2 | 42.6% | 0% | 29% | 13% | 14.8 | 100% Chinese | China |
| REVEAL, Menter *et al.*, 2008 | Placebo | 16 weeks | 398 | 45.4 | 65% | 94.1 kg | 18.8 | 25.6% | 13% | 22% | 28% | 18.4 | 90.2% Caucasian | USA and Canada |
|  | Adalimumab 40 mg Q2W |  | 814 | 44.1 | 67% | 92.3 kg | 19 | 25.8% | 12% | 23% | 28% | 18.1 | 91.2% Caucasian | USA and Canada |
| Asahina *et al.*, 2010 | Placebo | 16 weeks | 46 | 43.9 | 89% | 71.3 kg | 29.1 | 46.7% | NR | NR | NR | 15.5 | 100% Japanese | Japan |
|  | Adalimumab 40 mg Q2W (no loading dose) |  | 38 | 47.8 | 84% | 69.7 kg | 25.4 | 43.3% | NR | NR | NR | 14.2 | 100% Japanese | Japan |
|  | Adalimumab 40 mg Q2W (with loading dose) |  | 43 | 44.2 | 81% | 67.4 kg | 30.2 | 48.3% | NR | NR | NR | 14 | 100% Japanese | Japan |
|  | Adalimumab 80 mg Q2W |  | 42 | 43.5 | 83% | 72 kg | 28.3 | 46.1% | NR | NR | NR | 11.6 | 100% Japanese | Japan |
| Gordon *et al.*, 2006 (M02-528) | Placebo | 12 weeks | 52 | 43 | 65% | 94 kg | 16 | 28% | NR | NR | 31% | 19 | 92% Caucasian | USA and Canada |
|  | Adalimumab 40 mg QW |  | 50 | 44 | 66% | 99 kg | 14.5 | 25% | NR | NR | 24% | 18 | 90% Caucasian | USA and Canada |
|  | Adalimumab 40 mg Q2W |  | 45 | 46 | 71% | 93 kg | 16.7 | 29% | NR | NR | 33% | 21 | 89% Caucasian | USA and Canada |
| X-PLORE, Gordon *et al.*, 2015 | Placebo | 16 weeks | 42 | 46.5 | 67% | 93.6 kg | 21.8 | 28% | 36% | 50% | 29% | 18 | 93% white | North America and Europe |
|  | Guselkumab 5 mg |  | 41 | 44 | 72% | 90.7 kg | 20.9 | 25% | 41% | 52% | 25% | 18.5 | 91% white | North America and Europe |
|  | Guselkumab 15 mg |  | 41 | 44 | 72% | 90.7 kg | 20.9 | 25% | 41% | 52% | 25% | 18.5 | 91% white | North America and Europe |
|  | Guselkumab 50 mg |  | 42 | 44 | 72% | 90.7 kg | 20.9 | 25% | 41% | 52% | 25% | 18.5 | 91% white | North America and Europe |
|  | Guselkumab 100 mg |  | 42 | 44 | 72% | 90.7 kg | 20.9 | 25% | 41% | 52% | 25% | 18.5 | 91% white | North America and Europe |
|  | Guselkumab 200 mg |  | 42 | 44 | 72% | 90.7 kg | 20.9 | 25% | 41% | 52% | 25% | 18.5 | 91% white | North America and Europe |
|  | Adalimumab 40mg Q2W |  | 43 | 50 | 70% | 91.6 kg | 20.2 | 27% | 60% | 40% | 26% | 19 | 91% white | North America and Europe |
| Bissonnette *et al.*, 2013 | Placebo | 16 weeks | 10 | 57.4 | 60% | 94.8 kg | 13.1 | 13% | NR | NR | NR | NR | 100% white | Canada |
|  | Adalimumab 40mg Q2W |  | 20 | 56.1 | 85% | 95.1 kg | 11.6 | 12% | NR | NR | NR | NR | 100% white | Canada |
| VOYAGE 1, Blauvelt *et al.*, 2017 | Placebo | 16 weeks | 174 | 44.9 | 68% | NR | 20.4 | 25.8% | 20% | 53% | 17% | 17.6 | 83.3% white, 13.2% Asian, 1.7% black | International |
|  | Guselkumab 100 mg Q8W |  | 329 | 43.9 | 73% | NR | 22.1 | 28.3% | 22% | 64% | 20% | 17.9 | 79.6% white, 15.5% Asian, 1.8% black | International |
|  | Adalimumab 40 mg Q2W |  | 334 | 42.9 | 75% | NR | 22.4 | 28.6% | 21% | 64% | 19% | 17 | 82.9% white, 14.1% Asian, 2.4% black | International |
| VOYAGE 2, Reich *et al.*, 2017 | Placebo | 16 weeks | 248 | 43.3 | 70% | NR | 21.5 | 28% | 22% | 60% | 19% | 17.9 | 83.1% white, 10.9% Asian, 3.2% black | International |
|  | Guselkumab 100 mg |  | 496 | 43.7 | 70% | NR | 21.9 | 28.5% | 20% | 67% | 18% | 17.9 | 82.3% white, 14.5% Asian, 1.2% black | International |
|  | Adalimumab 40 mg Q2W |  | 248 | 43.2 | 69% | NR | 21.7 | 29.1% | 20% | 64% | 18% | 17.6 | 80.6% white, 14.9% Asian, 2% black | International |
| PSOR-005, Papp *et al.*, 2012 (CORE) | Placebo | 16 weeks | 88 | 44.1 | 60% | 90.4 kg | 18.1 | 21% | NR | 44% | 19% | 19.6 | 94% white,  1% black,  5% Asian | USA and Canada |
|  | Apremilast 10 mg BID |  | 89 | 44.4 | 71% | 95.9 kg | 18.1 | 21.3% | NR | 53% | 23% | 18 | 92% white,  2% black,  3% Asian,  2% other | USA and Canada |
|  | Apremilast 20 mg BID |  | 87 | 44.6 | 63% | 89.9 kg | 18.5 | 20.7% | NR | 49% | 18% | 19.2 | 94% white,  1% black,  2% Asian,  2% other | USA and Canada |
|  | Apremilast 30 mg BID |  | 88 | 44.1 | 57% | 91.4 kg | 19.1 | 25% | NR | 53% | 24% | 19.2 | 91% white,  2% black,  5% Asian,  2% other | USA and Canada |
| ESTEEM 1, Papp *et al.*, 2015 | Placebo | 16 weeks | 282 | 46.5 | 69% | 93.7 kg | 19.4 | 25.3% | 28% | 36% | NR | 18.7 | 88.7% white, 5.7% Asian, 3.5% black, 2.1% other | International |
|  | Apremilast 30 mg BID |  | 562 | 45.8 | 67% | 93.2 kg | 18.7 | 24.4% | 29% | 38% | NR | 19.8 | 90.2% white, 5% Asian, 3.2% black, 1.6% other | International |
| ESTEEM 2, Paul *et al.*, 2015 | Placebo | 16 weeks | 137 | 45.7 | 73% | 90.5 kg | 20 | 27.6% | 32% | 39% | NR | 18.7 | 93.4% white | USA, Canada and Europe |
|  | Apremilast 30 mg BID |  | 274 | 45.3 | 64% | 91.4 kg | 18.9 | 25.5% | 34% | 39% | NR | 17.9 | 91.2% white | USA, Canada and Europe |
| Ohtsuki *et al.*, 2017 | Placebo | 16 weeks | 84 | 48.3 | 74% | 68.5 kg | 19.9 | 28% | 5% | 26% | NR | 12.4 | 100% Japanese | Japan |
|  | Apremilast 20 mg BID |  | 85 | 52.2 | 81% | 71.2 kg | 22.1 | 32% | 4% | 40% | NR | 12.6 | 100% Japanese | Japan |
|  | Apremilast 30 mg BID |  | 85 | 51.7 | 84% | 70.1 kg | 21.6 | 30.7% | 2% | 31% | NR | 13.9 | 100% Japanese | Japan |
| LIBERATE, Reich *et al.*, 2016 | Placebo | 16 weeks | 84 | 43.4 | 70% | 89.5 kg | 19.4 | 27.3% | 0% | 83% | NR | 16.6 | 95.2% white, 2.4% Asian, 1.2% black, 1.2% other | International |
|  | Apremilast 30 mg BID |  | 83 | 46 | 59% | 88.5 kg | 19.3 | 27.1% | 0% | 80% | NR | 19.7 | 95.2% white, 3.6% black, 1.2% other | International |
|  | Etanercept 50 mg QW |  | 83 | 47 | 59% | 88.1 kg | 20.3 | 28.4% | 0% | 70% | NR | 18.1 | 90.4% white, 1.2% Asian, 6% black, 2.4% other | International |
| Leonardi *et al.*, 2003 | Placebo | 12 weeks | 166 | 45.6 | 63% | NR | 18.3 | 29% | NR | NR | 22 | 18.4 | 90% white | USA |
|  | Etanercept 25 mg BIW |  | 160 | 44.4 | 74% | NR | 18.2 | 28% | NR | NR | 22 | 19.3 | 85% white | USA |
|  | Etanercept 50 mg QW |  | 162 | 45.4 | 67% | NR | 18.5 | 29% | NR | NR | 22 | 18.5 | 85% white | USA |
|  | Etanercept 50 mg BIW |  | 164 | 44.8 | 65% | NR | 18.4 | 30% | NR | NR | 22 | 18.6 | 87% white | USA |
| Gottlieb *et al.*, 2003 | Placebo | 12 weeks | 55 | 46.5 | 67% | 90.7 kg | 19.5 | 34% | NR | 36% | 35% | 20 | 95% white,  2% black,  2% Hispanic, 2% other | USA |
|  | Etanercept 25 mg BIW |  | 57 | 48.2 | 58% | 91.8 kg | 17.8 | 30% | NR | 39% | 28% | 23 | 89% white,  2% black,  7% Hispanic, 2% other | USA |
| Papp *et al.*, 2005 | Placebo | 12 weeks | 193 | 44.8 | 64% | NR | 18.6 | 27% | NR | 39% | 26% | 19 | 91% white | USA, Canada and Europe |
|  | Etanercept 25mg BIW |  | 196 | 45.4 | 65% | NR | 19.1 | 29% | NR | 35% | 28% | 22 | 92% white | USA, Canada and Europe |
|  | Etanercept 50mg BIW |  | 194 | 45.2 | 67% | NR | 19.5 | 29% | NR | 38% | 26% | 20 | 89% white | USA, Canada and Europe |
| Van de Kerkhof *et al.*, 2008 | Placebo | 12 weeks | 46 | 43.6 | 54% | 79.1 kg | 21 | 30.3% | NR | 48% | 11% | 17 | NR | Europe |
|  | Etanercept 50mg QW |  | 96 | 45.9 | 62% | 83.4 kg | 21.4 | 26.5% | NR | 49% | 16% | 19 | NR | Europe |
| Bagel *et al.*, 2012 | Placebo | 12 weeks | 62 | 42 | 58% | NR | 15.2 | 15% | 11% | NR | NR | 12 | 75.8% white or Caucasian, 9.7% Black, 9.7% Hispanic or Latino, 3.2% Asian, 1.6% other | North America |
|  | Etanercept 50mg BIW |  | 62 | 39 | 53% | NR | 15.5 | 16% | 10% | NR | NR | 18 | 69.4% White or Caucasian, 8.1% black, 12.9% Hispanic or Latino,  4.8% Asian, 4.8% other | North America |
| Bachelez *et al.*, 2015 | Placebo | 12 weeks | 107 | 46 | 66% | 80.2 kg | 19.5 | 26% | 11% | 82% | 24% | 17 | 84% white,  7% Asian,  8% other | International (other than USA and Canada) |
|  | Etanercept 50mg BIW |  | 335 | 42 | 70% | 82 kg | 19.4 | 25% | 11% | 83% | 21% | 18 | 87% white,  6% Asian,  7% other | International (other than USA and Canada) |
| Tyring *et al.*, 2006 | Placebo | 12 weeks | 307 | 45.6 | 70% | 91 kg | 18.1 | 27% | NR | NR | 33% | 20 | 88% white | USA and Canada |
|  | Etanercept 50mg BIW |  | 311 | 45.8 | 65% | 92.6 kg | 18.3 | 27% | NR | NR | 35% | 20 | 90% white | USA and Canada |
| PRISTINE, Strohal *et al.*, 2013 | Etanercept 50mg QW | 12 weeks | 137 | 43.9 | 74% | 86.6 kg | 20.9 | 33% | 0% | NR | 29% | 17 | 62.8% white, 24.1% Asian, 13.1% other | Europe, Latin America and Asia |
|  | Etanercept 50mg BIW |  | 136 | 44 | 65% | 83.7 kg | 21.4 | 33% | 0% | NR | 33% | 18 | 64.7% white, 22.8% Asian, 12.5% other | Europe, Latin America and Asia |
| M10-114, Gottlieb *et al.*, 2011 | Placebo | 12 weeks | 68 | 44 | 69% | 96.5 kg | 18.5 | 24% | 15% | 28% | 21% | 19 | 95.6% Caucasian | USA |
|  | Etanercept 50mg BIW |  | 141 | 43.1 | 70% | 94.5 kg | 19.4 | 24% | 14% | 26% | 23% | 17 | 90.1% Caucasian | USA |
| M10-315, Strober *et al.*, 2011 | Placebo | 12 weeks | 72 | 45 | 64% | 92.9 kg | 18.3 | 22% | 4% | 28% | 21% | 16 | 93.1% Caucasian | USA |
|  | Etanercept 50mg BIW |  | 139 | 45.2 | 61% | 96.9 kg | 18.5 | 25% | 8% | 32% | 33% | 15 | 91.4% Caucasian | USA |
| reSURFACE2 | Placebo | 12 weeks | 156 | 46.4 | 72% | 88.7 kg | 20 | 31% | 13% | NR | NR | NR | 92% white,  2% Asian,  6% other | Europe, Israel and USA |
|  | Tildrakizumab 100 mg |  | 307 | 44.6 | 72% | 89.4 kg | 20.5 | 34% | 13% | NR | NR | NR | 91% white,  3% Asian,  6% other | Europe, Israel and USA |
|  | Tildrakizumab 200 mg |  | 314 | 44.6 | 72% | 88.4 kg | 19.8 | 32% | 12% | NR | NR | NR | 90% white,  4% Asian,  5% other | Europe, Israel and USA |
|  | Etanercept 50 mg BIW |  | 313 | 45.8 | 71% | 88 kg | 20.2 | 32% | 12% | NR | NR | NR | 92% white,  3% Asian,  4% other | Europe, Israel and USA |
| PIECE, De Vries *et al.*, 2016 | Etanercept 50mg BIW | 12 weeks | 23 | 42.4 | 57% | NR | 15.9 | 21% | 22% | 100% | 13% | 18 | NR | The Netherlands |
|  | Infliximab 5mg/kg |  | 25 | 45.9 | 72% | NR | 17.8 | 28% | 12% | 96% | 8% | 22 | NR | The Netherlands |
| Yang *et al.*, 2012 | Placebo | 10 weeks | 45 | 40.1 | 78% | 67.4 kg | 25.3 | NR | NR | 100% | NR | 16 | 100% Chinese | China |
|  | Infliximab 5mg/kg |  | 84 | 39.4 | 71% | 68.2 kg | 23.9 | NR | NR | 100% | NR | 16 | 100% Chinese | China |
| EXPRESS, Reich *et al.*, 2005 | Placebo | 10 weeks | 77 | 43.8 | 79% | 89.3 kg | 22.8 | 34% | NR | NR | 29% | 17 | NR | International |
|  | Infliximab 5mg/kg |  | 301 | 42.6 | 69% | 85.9 kg | 22.9 | 34% | NR | NR | 31% | 19 | NR | International |
| Chaudhari *et al.*, 2001 | Placebo | 10 weeks | 11 | 45 | 73% | 85 kg | 20.3 | NR | 0% | NR | NR | NR | NR | USA |
|  | Infliximab 5mg/kg |  | 11 | 51 | 64% | 87 kg | 22.1 | NR | 0% | NR | NR | NR | NR | USA |
|  | Infliximab 10mg/kg |  | 11 | 35 | 73% | 96 kg | 26.6 | NR | 0% | NR | NR | NR | NR | USA |
| SPIRIT, Gottlieb *et al.*, 2004 | Placebo | 10 weeks | 51 | 45 | 61% | NR | 18 | 26% | 31% | 82% | 33% | 16 | NR | USA |
|  | Infliximab 3mg/kg |  | 99 | 45 | 71% | NR | 20 | 29% | 32% | 87% | 32% | 18 | NR | USA |
|  | Infliximab 5mg/kg |  | 99 | 44 | 74% | NR | 20 | 25% | 33% | 89% | 29% | 16 | NR | USA |
| EXPRESS II, Menter *et al.*, 2007 | Placebo | 10 weeks | 208 | 44.4 | 69% | 91.1 kg | 19.8 | 28% | 13% | NR | 26% | 18 | 90.9% Caucasian | USA, Canada and Europe |
|  | Infliximab 3mg/kg |  | 313 | 43.4 | 66% | 92 kg | 20.1 | 28% | 16% | NR | 28% | 18 | 93% Caucasian | USA, Canada and Europe |
|  | Infliximab 5mg/kg |  | 314 | 44.5 | 65% | 92.2 kg | 20.4 | 29% | 14% | NR | 28% | 19 | 93.3% Caucasian | USA, Canada and Europe |
| Torii *et al.*, 2010 | Placebo | 10 weeks | 19 | 43.3 | 74% | 69.7 kg | 33.1 | 50% | NR | 95% | 37% | 11 | 100% Japanese | Japan |
|  | Infliximab 5mg/kg |  | 35 | 46.9 | 63% | 68.5 kg | 31.9 | 46% | NR | 94% | 29% | 14 | 100% Japanese | Japan |
| RESTORE1, Barker *et al.*, 2011 | Infliximab 5mg/kg | 16 weeks | 653 | 44.1 | 67% | 84.5 kg | 21.4 | 32% | 8% | 61% | NR | 19 | 97% Caucasian | Europe |
|  | Methotrexate |  | 215 | 41.9 | 69% | 83.8 kg | 21.1 | 31% | 8% | 65% | NR | 17 | 98% Caucasian | Europe |
| UNCOVER-1, Gordon *et al.*, 2016 | Placebo | 12 weeks | 431 | 46 | 70% | 92 kg | 20 | 27% | 42% | 52% | NR | 20 | 93% white | International |
|  | Ixekizumab 80mg Q4W |  | 432 | 46 | 67% | 92 kg | 20 | 27% | 39% | 49% | NR | 19 | 91.9% white | International |
|  | Ixekizumab 80mg Q2W |  | 433 | 45 | 67% | 92 kg | 20 | 28% | 40% | 57% | NR | 20 | 92.6% white | International |
| UNCOVER-2, Griffiths *et al.*, 2015 | Placebo | 12 weeks | 168 | 45 | 71% | 92 kg | 21 | 27% | 26% | 48% | NR | 19 | 88.7% white | International |
|  | Etanercept 50mg BIW |  | 358 | 45 | 66% | 93 kg | 19 | 25% | 21% | 48% | NR | 19 | 93.5% white | International |
|  | Ixekizumab 80mg Q4W |  | 347 | 45 | 70% | 93 kg | 20 | 27% | 25% | 51% | NR | 19 | 91.8% white | International |
|  | Ixekizumab 80mg Q2W |  | 351 | 45 | 63% | 89 kg | 19 | 25% | 24% | 51% | NR | 18 | 94.3% white | International |
| UNCOVER-3, Griffiths *et al.*, 2015 | Placebo | 12 weeks | 193 | 46 | 71% | 91 kg | 21 | 29% | 17% | 43% | NR | 18 | 91.2% white | International |
|  | Etanercept 50mg BIW |  | 382 | 46 | 70% | 92 kg | 21 | 28% | 16% | 48% | NR | 18 | 91.9% white | International |
|  | Ixekizumab 80mg Q4W |  | 386 | 46 | 67% | 91 kg | 21 | 28% | 15% | 47% | NR | 18 | 93.3% white | International |
|  | Ixekizumab 80mg Q2W |  | 385 | 46 | 66% | 90 kg | 21 | 28% | 15% | 44% | NR | 18 | 93.8% white | International |
| IXORA-S, Reich *et al.* 2017 | Ixekizumab 80 mg Q2W | 12 weeks | 136 | 42.7 | 66% | 85.8 kg | 19.9 | 27% | 13% | 93% | NR | 18 | 93.3% white | International? 13 countries |
|  | Ustekinumab (45 mg if <100 kg; 90 mg if >100 kg) |  | 166 | 44 | 68% | 89.4 kg | 19.8 | 28% | 15% | 92% | NR | 18 | 95.7% white | International? 13 countries |
| FEATURE, Blauvelt *et al.*, 2015 | Placebo | 12 weeks | 59 | 46.5 | 66% | 88.4 kg | 21.1 | 32% | 44% | 49% | NR | 20 | 96.6% white | North America and Europe |
|  | Secukinumab 150mg |  | 59 | 46 | 68% | 93.7 kg | 20.5 | 31% | 48% | 66% | NR | 20 | 86.4% white | North America and Europe |
|  | Secukinumab 300mg |  | 59 | 45.1 | 64% | 92.6 kg | 20.7 | 33% | 39% | 34% | NR | 18 | 91.5% white | North America and Europe |
| ERASURE, Langley *et al.*, 2014 | Placebo | 12 weeks | 248 | 45.4 | 69% | 89.7 kg | 21.4 | 30% | 29% | 44% | 27% | 17 | 71% white, 18.5% Asian, 10.5% other/unknown | International |
|  | Secukinumab 150mg |  | 245 | 44.9 | 69% | 87.1 kg | 22.3 | 33% | 30% | 51% | 19% | 18 | 69.8% white, 22% Asian, 8.2% other/unknown | International |
|  | Secukinumab 300mg |  | 245 | 44.9 | 69% | 88.8 kg | 22.5 | 33% | 29% | 52% | 23% | 17 | 69.8% white, 21.2% Asian, 9% other/unknown | International |
| FIXTURE, Langley *et al.*, 2014 | Placebo | 12 weeks | 326 | 44.1 | 73% | 82 kg | 24.1 | 35% | 11% | 61% | 15% | 17 | 66.9% white, 22.1% Asian, 11% other/unknown | International |
|  | Etanercept 50mg BIW |  | 326 | 43.8 | 71% | 84.6 kg | 23.2 | 34% | 14% | 63% | 14% | 16 | 67.2% white, 22.7% Asian, 10.1% other/unknown | International |
|  | Secukinumab 150mg |  | 327 | 45.4 | 72% | 83.6 kg | 23.7 | 35% | 14% | 61% | 15% | 17 | 67% white, 22% Asian, 11% other/unknown | International |
|  | Secukinumab 300mg |  | 327 | 44.5 | 69% | 83 kg | 23.9 | 34% | 12% | 60% | 15% | 16 | 68.5% white, 22.3% Asian, 9.2% other/unknown | International |
| JUNCTURE, Paul *et al.*, 2015 | Placebo | 12 weeks | 61 | 43.7 | 62% | 90.2 kg | 19.4 | 26% | 21% | 48% | 20% | 20 | 96.7% Caucasian | International |
|  | Secukinumab 150mg |  | 61 | 43.9 | 67% | 93.7 kg | 22 | 30% | 25% | 51% | 26% | 21 | 95.1% Caucasian | International |
|  | Secukinumab 300mg |  | 60 | 46.6 | 77% | 91 kg | 18.9 | 26% | 25% | 50% | 23% | 21 | 93.3% Caucasian | International |
| CLEAR, Thaci *et al.*, 2015 | Secukinumab 300mg | 16 weeks | 337 | 45.2 | 68% | 87.4 kg | 21.7 | 33% | 14% | 65% | 21% | 20 | 88.7% Caucasian, 11.3% other | International (134 sites) |
|  | Ustekinumab (45 mg if <100 kg; 90 mg if >100 kg) |  | 339 | 44.6 | 74% | 87.2 kg | 21.5 | 32% | 13% | 66% | 16% | 16 | 85% Caucasian, 15% other | International (134 sites) |
| PEARL, Tsai *et al.*, 2011 | Placebo | 12 weeks | 60 | 40.4 | 88% | 74.6 kg | 22.9 | 36% | 15% | 72% | 12% | 14 | 50% Taiwanese/Chinese,  50% Korean | Korea and Taiwan |
|  | Ustekinumab 45mg |  | 61 | 40.9 | 82% | 73.1 kg | 25.2 | 42% | 21% | 71% | 16% | 12 | 49% Taiwanese/Chinese,  51% Korean | Korea and Taiwan |
| PHOENIX-1, Leonardi *et al.*, 2008 | Placebo | 12 weeks | 255 | 44.8 | 72% | 94.2 kg | 20.4 | 28% | 50% | 56% | 35% | 20 | NR | USA, Canada and Belgium |
|  | Ustekinumab 45mg |  | 255 | 44.8 | 69% | 93.7 kg | 20.5 | 27% | 53% | 55% | 29% | 20 | NR | USA, Canada and Belgium |
|  | Ustekinumab 90mg |  | 256 | 46.2 | 68% | 93.8 kg | 19.7 | 25% | 51% | 55% | 37% | 20 | NR | USA, Canada and Belgium |
| PHOENIX-2, Papp *et al.*, 2008 | Placebo | 12 weeks | 410 | 47 | 69% | 91.1 kg | 19.4 | 26% | 39% | 59% | 26% | 21 | NR | Europe and North America |
|  | Ustekinumab 45mg |  | 409 | 45.1 | 69% | 90.3 kg | 19.4 | 26% | 38% | 55% | 26% | 19 | NR | Europe and North America |
|  | Ustekinumab 90mg |  | 411 | 46.6 | 67% | 91.5 kg | 20.1 | 27% | 37% | 55% | 23% | 20 | NR | Europe and North America |
| LOTUS, Zhu *et al.*, 2013 | Placebo | 12 weeks | 162 | 39.2 | 76% | 70 kg | 22.7 | 35% | 7% | 43% | 9% | 14 | 100% Chinese | China |
|  | Ustekinumab 45mg |  | 160 | 40.1 | 78% | 69.9 kg | 23.2 | 35% | 12% | 39% | 9% | 15 | 100% Chinese | China |
| ACCEPT, Griffiths *et al.*, 2010 | Etanercept 50mg BIW | 12 weeks | 347 | 45.7 | 71% | 90.8 kg | 18.6 | 24% | 12% | 57% | 27% | 19 | 91% white | International |
|  | Ustekinumab 45mg |  | 209 | 45.1 | 64% | 90.4 kg | 20.5 | 27% | 12% | 62% | 30% | 19 | 92% white | International |
|  | Ustekinumab 90mg |  | 347 | 44.8 | 67% | 91 kg | 19.9 | 26% | 10% | 52% | 27% | 19 | 89% white | International |
| Igarashi *et al.*, 2012 | Placebo | 12 weeks | 32 | 49 | 84% | 71.2 kg | 30.3 | 50% | 0% | 66% | 3% | 16 | 100% Japanese | Japan |
|  | Ustekinumab 45mg |  | 64 | 45 | 83% | 73.2 kg | 30.1 | 47% | 2% | 73% | 9% | 16 | 100% Japanese | Japan |
|  | Ustekinumab 90mg |  | 62 | 44 | 76% | 71.1 kg | 28.7 | 47% | 0% | 84% | 11% | 17 | 100% Japanese | Japan |
| BRIDGE, Mrowietz *et al.,* 2017 | Placebo | 16 weeks | 137 | 44 | 68% | NR | 16.2 | 22% | TNF:2%, IL:0% | MTX:10%, CIC:6%, ACI:7% | NR | NR | 100% white | Austria, Germany, the Netherlands and Poland |
|  | Dimethyl Fumarate |  | 279 | 44 | 62% | NR | 16.3 | 22% | TNF:2.5%, IL:1% | MTX:7%, CIC:4%, ACI:3% | NR | NR | 98.6% white, 0.4% black, 0.4% Asian, 0.7% other | Austria, Germany, the Netherlands and Poland |
|  | Fumaderm |  | 283 | 45 | 65% | NR | 16.4 | 21% | TNF:2%, IL:1% | MTX:14%, CIC:3%, ACI:5% | NR | NR | 98.9% white, 1.1% Asian | Austria, Germany, the Netherlands and Poland |
| Caproni *et al.*, 2009 | Etanercept 50mg BIW | 12 weeks | 30 | NR | 43% | NR | 21.54 | NR | NR | NR | NR | NR | NR | Italy |
|  | Acitretin |  | 30 | NR | 37% | NR | 22.25 | NR | NR | NR | NR | NR | NR | Italy |
| Gisondi *et al.*, 2008 | Etanercept 25mg BIW | 12 weeks | 22 | 55.3 | 55% | 79.5 kg | 11 | 13% | 0% | NR | 0% | 24 | NR | Italy |
|  | Etanercept 25mg once per week + Acitretin (0.4mg/kg-1) |  | 18 | 53.4 | 50% | 77.5 kg | 11.9 | 13% | 0% | NR | 0% | 19 | NR | Italy |
|  | Acitretin (0.4mg/kg-1) |  | 20 | 55 | 60% | 78.4 kg | 10.4 | 11% | 0% | NR | 0% | 19 | NR | Italy |
| Meffert *et al.* | Cyclosporin 2.5 mg/kg/day | 10 weeks | 44 | 38.2 | 64% | NR | NR | NR | 60.90% | NR | NR | NR | NR | Germany |
|  | Cyclosporin 1.25 mg/kg/day |  | 41 |  |  | NR | NR | NR |  | NR | NR | NR | NR | Germany |
|  | PBO |  | 43 |  |  | NR | NR | NR |  | NR | NR | NR | NR | Germany |
| Papp *et al.*, 2015 | Tildrakizumab 100 mg | 16 weeks | 89 | 45.5 | 85% | NR | NR | NR | 17% prior TNF inhibitor use |  | 17% | NR | 82% white, 18% non-white | USA, Canada, Japan and Europe |
|  | Placebo |  | 46 | 45.9 | 83% | NR | NR | NR | 26% prior TNF inhibitor use |  | 24% | NR | 76% white, 24% non-white | USA, Canada, Japan and Europe |
|  | Tildrakizumab 5 mg |  | 42 | 43.2 | 74% | NR | NR | NR | 21% prior TNF inhibitor use |  | 19% | NR | 74% white, 26% non-white | USA, Canada, Japan and Europe |
|  | Tildrakizumab 25 mg |  | 92 | 46.3 | 65% | NR | NR | NR | 18% prior TNF inhibitor use |  | 16% | NR | 85% white, 15% non-white | USA, Canada, Japan and Europe |
|  | Tildrakizumab 200 mg |  | 86 | 43.2 | 76% | NR | NR | NR | 17% prior TNF inhibitor use |  | 17% | NR | 85% white, 15% non-white | USA, Canada, Japan and Europe |
| Re-SURFACE 1 | Placebo | 12 weeks | 155 | 47.9 | 65% | 87.5 kg | 19.3 | 29.6% | 23% | 35% | NR | NR | 65% white, 27% Asian, 8% other | Australia, Canada, Japan, UK and USA |
|  | Tildrakizumab 100mg |  | 309 | 46.4 | 67% | 88.5 kg | 20 | 29.7% | 23% | 23% | NR | NR | 70% white, 23% Asian, 7% other | Australia, Canada, Japan, UK and USA |
|  | Tildrakizumab 200mg |  | 308 | 46.9 | 73% | 88.9 kg | 20.7 | 30.9% | 23% | 23% | NR | NR | 68% white, 27% Asian, 5% other | Australia, Canada, Japan, UK and USA |
| ultIMMA-1 (press release) | Placebo | 16 weeks | 102 | NR | NR | NR | NR | NR | NR | NR | NR | NR | NR | NR |
|  | Risankizumab 150mg |  | 304 | NR | NR | NR | NR | NR | NR | NR | NR | NR | NR | NR |
|  | Ustekinumab 45/90mg |  | 100 | NR | NR | NR | NR | NR | NR | NR | NR | NR | NR | NR |
| ultIMMA-2 (press release) | Placebo | 16 weeks | 98 | NR | NR | NR | NR | NR | NR | NR | NR | NR | NR | NR |
|  | Risankizumab 150mg |  | 294 | NR | NR | NR | NR | NR | NR | NR | NR | NR | NR | NR |
|  | Ustekinumab 45/90mg |  | 99 | NR | NR | NR | NR | NR | NR | NR | NR | NR | NR | NR |
| METOP, Warren *et al.* | Placebo | 16 weeks | 29 | 44.4 | 86% | 95.9 kg | 15.4 | 19.6% | 3% | 24% | 7% | 14 | 100% white | Europe & UK |
|  | Methotrexate |  | 91 | 45.9 | 71% | 92.4 kg | 15.4 | 20% | 5% | 32% | 12% | 21 | 98% white,  2% Asian | Europe & UK |
| Krueger *et al.* | PBO | 16 weeks | 64 | 44 | 72% | 92.8 kg | 19.9 | 26.6% | NR | 61% | 19% | 17 | NR | International |
|  | Ustekinumab 45mg (4 weekly doses) |  | 64 | 45 | 61% | 92.8 kg | 18.9 | 27.4% | NR | 72% | 19% | 20 | NR | International |
|  | Ustekinumab 45 mg |  | 64 | 46 | 59% | 94.3 kg | 19 | 28.5% | NR | 61% | 20% | 19 | NR | International |
|  | Ustekinumab 90mg (4 weekly doses) |  | 64 | 44 | 81% | 91.9 kg | 19 | 27.4% | NR | 55% | 20% | 17 | NR | International |
|  | Ustekinumab 90 mg |  | 64 | 46 | 73% | 92.9 kg | 18.8 | 26.3% | NR | 58% | 19% | 18 | NR | International |
| Reich *et al.,* 2012 | PBO | 12 weeks | 59 | 43.3 | 63% | 79.2 kg | 22.6 | 30.1% | 24% anti-TNF | NR | NR | 20 | 97% Caucasian | France and Germany |
|  | CZP 200 mg Q2W |  | 59 | 43.3 | 75% | 84.4 kg | 21.4 | 26.7% | 22% anti-TNF | NR | NR | 21 | 97% Caucasian | France and Germany |
|  | CZP 400 mg Q2W |  | 58 | 43.6 | 72% | 83.1 kg | 22 | 28.4% | 24% anti-TNF | NR | NR | 20 | 100% Caucasian | France and Germany |
| CIMPACT, 2018 | PBO | Week 16 | 57 | 46.5 | 60% | 93.7 kg | 19.1 | 24.3% | 19.30% | NR | 21.10% | 19 | 100% white | North America and Europe |
|  | CZP 200 mg Q2W |  | 165 | 46.7 | 69% | 89.7 kg | 21.4 | 28.1% | 26.70% | NR | 16.40% | 19.5 | 95.8% white | North America and Europe |
|  | CZP 400 mg Q2W |  | 167 | 45.4 | 64% | 86.3 kg | 20.8 | 27.6% | 28.70% | NR | 14.40% | 18 | 97% white | North America and Europe |
|  | Etanercept 50 mg | Week 12 | 170 | 44.6 | 75% | 88.6 kg | 21 | 27.5% | 30% | NR | 15.90% | 17 | 95.9% white | North America and Europe |
| CIMPASI 1, 2018 | PBO | Week 16 | 51 | 47.9 | 69% | 95.2 kg | 19.8 | 26.1% | 29.40% | 41.20% | 7.80% | 18.5 | 88.2% white | North America and Europe |
|  | CZP 200 mg Q2W |  | 95 | 44.5 | 71% | 92.6 kg | 20.1 | 25.4% | 31.60% | 37.90% | 10.50% | 17 | 91.6% white | North America and Europe |
|  | CZP 400 mg Q2W |  | 88 | 43.6 | 68% | 92.2 kg | 19.6 | 24.1% | 33% | 36.30% | 17% | 18 | 89.8% white | North America and Europe |
| CIMPASI 2, 2018 | PBO | Week 16 | 49 | 43.3 | 53% | 87.1 kg | 17.3 | 20% | 28.60% | 44.90% | 18.40% | 15 | 89.8% white | North America and Europe |
|  | CZP 200 mg Q2W |  | 91 | 46.7 | 64% | 97.8 kg | 18.4 | 21.4% | 35.20% | 36.20% | 24.20% | 19 | 94.5% white | North America and Europe |
|  | CZP 400 mg Q2W |  | 87 | 46.4 | 49% | 91.8 kg | 19.5 | 23.1% | 34.50% | 37.90% | 29.90% | 19 | 93.1% white | North America and Europe |
| UNVEIL (ongoing study) | Placebo | Week 16 | 73 | 51.1 | 56% | 89.6 kg | 8 | 7% | 0% | 0% | NR | 14 | NR | USA |
|  | Apremilast 30mg BID |  | 148 | 48.6 | 50% | 87.5 kg |  |  | 0% | 0% | NR | 18 | NR | USA |
