# Supplementary material for: Patient characteristics as effect modifiers for psoriasis biologic treatment response: an assessment using network meta-analysis subgroups
Source: Syst Rev. 2020 Jun 5;9:132. doi: 10.1186/s13643-020-01395-6 (PMC7275463; doi:10.1186/s13643-020-01395-6)
Supplement: Supplementary file 2 — Additional file 2:. Absolute probabilities of achieving PASI 75 across the five networks [file 13643_2020_1395_MOESM2_ESM.docx]

**Additional file 2: Absolute probabilities of achieving PASI 75 across the five networks**

| **Treatment** | **Absolute probability of achieving PASI 75**  **(95% CrI)** | | | | |
| --- | --- | --- | --- | --- | --- |
|  | **All licensed doses** | **No previous biologic use (<25%)** | **PASI score ≤25** | **Weight ≤90kg** | **≥90% white patients** |
| Adalimumab 40 mg | 0.65  (0.58-0.72) | 0.63  (0.55-0.71) | 0.66  (0.60-0.72) | 0.66  (0.54-0.77) | 0.67  (0.57-0.76) |
| Brodalumab 210 mg | 0.84  (0.79-0.89) | 0.84  (0.76-0.89) | 0.84  (0.80-0.88) | 0.84  (0.78-0.89) | 0.84  (0.78-0.89) |
| Certolizumab 200 mg | 0.60  (0.49-0.70) | 0.71  (0.43-0.91) | 0.61  (0.52-0.70) | 0.59  (0.48-0.70) | 0.62  (0.51-0.71) |
| Certolizumab 400 mg | 0.67  (0.57-0.76) | 0.80  (0.55-0.95) | 0.68  (0.59-0.76) | 0.66  (0.54-0.75) | 0.68  (0.58-0.77) |
| Etanercept 25 mg | 0.37  (0.28-0.48) | - | 0.39  (0.31-0.47) | - | 0.40  (0.29-0.53) |
| Etanercept 50 mg / week (QW) | 0.56  (0.44-0.68) | 0.26  (0.18-0.36) | 0.31  (0.23-0.40) | 0.28  (0.20-0.37) | 0.36  (0.11-0.80) |
| Etanercept 50 mg twice per week (BIW) | 0.50  (0.44-0.55) | 0.48  (0.41-0.55) | 0.53  (0.48-0.58) | 0.50  (0.43-0.57) | 0.52  (0.45-0.59) |
| Guselkumab 100 mg | 0.86  (0.81-0.91) | 0.85  (0.78-0.89) | 0.85  (0.80-0.89) | - | 0.78  (0.55-0.91) |
| Infliximab 5 mg | 0.81  (0.73-0.87) | 0.86  (0.75-0.93) | 0.78  (0.71-0.84) | 0.70  (0.59-0.80) | 15.38  (13.41-17.36) |
| Ixekizumab 80mg | 0.89  (0.86-0.92) | 0.88  (0.84-0.92) | 0.90  (0.87-0.92) | 0.87  (0.77-0.93) | 0.90  (0.87-0.93) |
| Risankizumab 150 mg | 0.84  (0.76-0.89) | - | - | - | - |
| Secukinumab 300mg | 0.83  (0.77-0.87) | 0.81  (0.75-0.87) | 0.83  (0.79-0.87) | 0.82  (0.76-0.86) | 0.95  (0.83-0.99) |
| Ustekinumab 45mg | 0.68  (0.61-0.75) | 0.62  (0.52-0.71) | 0.68  (0.62-0.74) | 0.66  (0.54-0.78) | 0.69  (0.61-0.77) |
| Ustekinumab 90mg | 0.73  (0.66-0.80) | 0.68  (0.57-0.77) | 0.74  (0.68-0.78) | 0.73  (0.53-0.87) | 0.74  (0.67-0.81) |
| Ustekinumab (45 mg or 90 mg) | 0.65  (0.58-0.72) | 0.66  (0.56-0.74) | 0.67  (0.60-0.73) | 0.66  (0.57-0.74) | 0.67  (0.58-0.75) |
| Tildrakizumab 100 mg | 0.77  (0.68-0.84) | 0.76  (0.68-0.83) | 0.80  (0.74-0.86) | 0.79  (0.71-0.85) | 0.82  (0.73-0.89) |
| Apremilast | 0.30  (0.23-0.39) | 0.19  (0.12-0.28) | 0.27  (0.21-0.33) | 0.20  (0.13-0.29) | 0.28  (0.21-0.36) |
| Dimethyl Fumarate | 0.15  (0.08-0.26) | 0.15  (0.09-0.25) | 0.15  (0.09-0.24) | - | 0.15  (0.08-0.25) |
| Fumaderm | 0.17  (0.09-0.28) | 0.17  (0.09-0.27) | 0.17  (0.11-0.26) | - | 0.17  (0.10-0.28) |
| Methotrexate | 0.32  (0.23-0.43) | 0.53  (0.34-0.72) | 0.34  (0.26-0.44) | 0.28  (0.19-0.41) | 0.32  (0.22-0.44) |
| Acitretin | 0.20  (0.08-0.40) | - | 0.22  (0.09-0.41) | - | 0.21  (0.09-0.43) |
| Cyclosporin 1.5mg | 0.40  (0.12-0.85) | - | - | - | 0.12  (0.02-0.53) |
| Cyclosporin 2.5 mg | 0.35  (0.10-0.82) | - | - | - | 0.36  (0.11-0.80) |
