# Supplementary material for: Patient characteristics as effect modifiers for psoriasis biologic treatment response: an assessment using network meta-analysis subgroups
Source: Syst Rev. 2020 Jun 5;9:132. doi: 10.1186/s13643-020-01395-6 (PMC7275463; doi:10.1186/s13643-020-01395-6)
Supplement: Supplementary file 3 — Additional file 3:. Table 1 Model fit for sensitivity analysis, Table 2 Median risk ratio and median ranks for the sensitivity analysis of patients with no previous biologic use including pre-2007 studies where prior exposure was not reported [file 13643_2020_1395_MOESM3_ESM.docx]

**Additional file 3**

**Table 1 Model fit for sensitivity analysis**

| **Measure of goodness of fit** | **Random effects (uniform prior)** | **Random effects**  **(log-normal prior)** | **Fixed effects** |
| --- | --- | --- | --- |

| **Network of patients with no previous biologic use (<25% had previous use) including pre-2007 studies where prior exposure was not reported** | | | |
| --- | --- | --- | --- |
| Residual deviance^2^ | 90.01 | 91.63 | 96.64 |
| pD | 67.47 | 64.38 | 59.61 |
| Deviance information criterion (DIC) | 157.49 | 156.01 | 156.25 |
| Between-study standard deviation, posterior median (95% credible interval) | 0.19 (0.04-0.37) | 0.14 (0.09-0.21) | - |

The random effects model with a log-normal prior distribution was chosen. The DIC was very similar between the random effects models and the fixed effects model, however the log-normal prior model had a smaller residual deviance than the fixed effects model and a smaller number of parameters than the uniform prior random effects model.

**Table 2 Median risk ratio and median ranks for the sensitivity analysis of patients with no previous biologic use including pre-2007 studies where prior exposure was not reported**

| **Treatment** | **Median risk ratio versus placebo – PASI 75**  **(95% CrI)** | **Median rank (95% CrI)** |
| --- | --- | --- |
|  | **No previous biologic use (<25%** | |
| Adalimumab | 12.46  (10.92-14.10) | 11 (8-12) |
| Apremilast | 3.74  (2.48-5.55) | 16 (15-17) |
| Brodalumab | 16.51  (14.73-18.34) | 4 (1-7) |
| Certolizumab 200 mg | 14.01  (8.59-18.26) | 8 (2-13) |
| Certolizumab 400 mg | 15.81  (10.89-19.19) | 6 (1-12) |
| DMF | 2.97  (1.76-4.95) | 17 (15-17) |
| Etanercept 25 mg | 7.24  (5.62-9.10) | 14 (14-15) |
| Etanercept 50 mg twice per week | 9.76  (8.69-10.96) | 13 (12-13) |
| Etanercept 50 mg once-weekly | 5.08  (3.54-6.98) | 15 (14-16) |
| Guselkumab 100 mg | 16.68  (15.06-18.40) | 4 (1-7) |
| Infliximab 5 mg | 17.07  (15.12-19.08) | 3 (1-7) |
| Ixekizumab 80mg | 17.52  (16.01-19.18) | 1 (1-4) |
| Risankizumab 150 mg | - | - |
| Secukinumab 300 mg | 16.14  (14.56-17.83) | 5 (2-8) |
| Tildrakizumab 100 mg | 15.21  (13.41-17.02) | 7 (4-9) |
| Ustekinumab 45 mg | 12.36  (10.54-14.25) | 11 (8-12) |
| Ustekinumab (45 mg or 90 mg) | 13.09  (11.19-15.00) | 10 (7-12) |
| Ustekinumab 90 mg | 13.56  (11.55-15.52) | 9 (7-12) |
| Fumaderm | 3.31  (1.98-5.40) | - |
| Methotrexate | 10.84  (6.73-14.41) | - |
| Acitretin | - | - |
| Cyclosporin 1.5 mg | - | - |
| Cyclosporin 2.5 mg | - | - |
| **Total number of treatments** | 19 | 17 |
