# Supplementary material for: Patient characteristics as effect modifiers for psoriasis biologic treatment response: an assessment using network meta-analysis subgroups
Source: Syst Rev. 2020 Jun 5;9:132. doi: 10.1186/s13643-020-01395-6 (PMC7275463; doi:10.1186/s13643-020-01395-6)
Supplement: Supplementary file 4 — Additional file 4:. Number of studies that report each continuous covariate for each treatment comparison [file 13643_2020_1395_MOESM4_ESM.docx]

**Additional file 4: Number of studies that report each continuous covariate for each treatment comparison**

| **Treatment comparison** | **Continuous covariates – no. of studies** | | | |
| --- | --- | --- | --- | --- |
|  | **Mean PASI score** | **Mean Weight** | **% White** | **% with previous biologic use** |
| ADA40mg vs GUS100mg | 3 | 1 | 3 | 3 |
| BROD210mg vs UST45mg90mg | 2 | 2 | 2 | 2 |
| Cyclosporin1.25mg vs Placebo | 0 | 0 | 0 | 0 |
| Cyclosporin2.5mg vs Cyclosporin1.25mg | 0 | 0 | 0 | 0 |
| Cyclosporin2.5mg vs Placebo | 0 | 0 | 0 | 0 |
| CZP200mg vs CZP400mg | 4 | 4 | 4 | 3 |
| DMF vs Fumaderm | 1 | 0 | 1 | 0 |
| ETN25mg vs Acitretin | 1 | 1 | 0 | 0 |
| ETN25mg vs ETN50mgBIW | 2 | 0 | 2 | 0 |
| ETN50mgBIW vs Acitretin | 1 | 0 | 0 | 0 |
| ETN50mgBIW vs INFLIX5mg | 1 | 0 | 0 | 1 |
| ETN50mgBIW vs IXE80mg | 2 | 2 | 2 | 2 |
| ETN50mgBIW vs SEC300mg | 1 | 1 | 1 | 1 |
| ETN50mgBIW vs UST45mg | 1 | 1 | 1 | 1 |
| ETN50mgBIW vs UST90mg | 1 | 1 | 1 | 1 |
| ETN50mgQW vs ETN50mgBIW | 1 | 1 | 1 | 1 |
| INFLIX5mg vs Methotrexate | 1 | 1 | 1 | 1 |
| IXE80mg vs UST45mg90mg | 1 | 1 | 1 | 1 |
| Methotrexate vs ADA40mg | 2 | 1 | 2 | 0 |
| Placebo vs ADA40mg | 9 | 7 | 9 | 5 |
| Placebo vs Apremilast30mg | 6 | 6 | 6 | 5 |
| Placebo vs BROD210mg | 5 | 5 | 5 | 4 |
| Placebo vs CZP200mg | 4 | 4 | 4 | 3 |
| Placebo vs CZP400mg | 4 | 4 | 4 | 3 |
| Placebo vs DMF | 1 | 0 | 1 | 0 |
| Placebo vs ETN25mg | 3 | 1 | 3 | 0 |
| Placebo vs ETN50mgBIW | 11 | 8 | 11 | 8 |
| Placebo vs Fumaderm | 1 | 0 | 1 | 0 |
| Placebo vs GUS100mg | 3 | 1 | 3 | 3 |
| Placebo vs INFLIX5mg | 6 | 5 | 3 | 3 |
| Placebo vs IXE80mg | 3 | 3 | 3 | 3 |
| Placebo vs Methotrexate | 2 | 2 | 2 | 1 |
| Placebo vs RIS150mg | 0 | 0 | 0 | 0 |
| Placebo vs SEC300mg | 4 | 4 | 4 | 4 |
| Placebo vs TIL100mg | 2 | 2 | 3 | 2 |
| Placebo vs UST45mg | 6 | 6 | 3 | 5 |
| Placebo vs UST45mg90mg | 2 | 2 | 2 | 2 |
| Placebo vs UST90mg | 4 | 4 | 1 | 3 |
| RIS150mg vs UST45mg90mg | 0 | 0 | 0 | 0 |
| SEC300mg vs UST45mg90mg | 1 | 1 | 1 | 1 |
| TIL100mg vs ETN50mgBIW | 1 | 1 | 1 | 1 |
| UST45mg vs UST90mg | 5 | 5 | 2 | 4 |
